# To Merge or not: The Early Onto‐ and Phylogenetic Origin of Co‐Representation

**DOI:** 10.1111/desc.70199

**Published:** 2026-04-26

**Authors:** Fabia M. Miss, Luxshiha Santharuban, Erik P. Willems, Judith M. Burkart

**Affiliations:** ^1^ Department of Evolutionary Anthropology University of Zurich Zurich Switzerland; ^2^ Center for the Interdisciplinary Study of Language Evolution ISLE University of Zurich Zurich Switzerland

**Keywords:** cooperative breeding, coordination smoother, co‐representation, evolution of cooperation, inhibitory control, mutual gaze, nonhuman primates, ontogeny, theory of mind development

## Abstract

**ABSTRACT:**

The origin of co‐representation during joint action poses a puzzle: It apparently only emerges around the age of four in humans, suggesting it is cognitively demanding, but has also been demonstrated in several nonhuman primate species whose cognitive skills do not match human four‐year‐olds. We therefore reassessed co‐representation in 2–4‐year‐old human children (*n* = 38, 11 females) with a directly comparable, nonverbal task previously applied to nonhuman primates. Co‐representation was already present and strongest in the youngest children, not constrained by Theory of Mind and inhibitory control skills, and weaker in children than in nonhuman primates. Together, this suggests co‐representation may be an early default mode of processing joint action. However, species differed in the flexibility to adjust when to merge perspectives by co‐representing, and when not. Children and the cooperatively breeding marmosets were most flexible and relied on coordination smoothers to achieve this (marmosets: mutual gaze; children: mutual gaze and communication).

**Summary:**

- Co‐representation, that is the merging of perspectives, is present in the joint Simon task in 2‐year‐olds and decreases with age
- Co‐representation is weaker in children than in nonhuman primates
- Cooperation success requires flexibly switching between merging or not
- Children and highly cooperative nonhuman primate species rely on mutual gaze as coordination smoothers for switching
- Co‐representation is likely an early default mode of processing joint actions

## 1 Introduction

Extensive cooperation and joint action are hallmarks of the human species. Intriguingly, a large body of evidence (initiated by the seminal work on the joint Simon effect by Sebanz et al. 2003, reviewed in Miss et al. 2022) has shown that when engaged in a joint task, humans represent not only their own task and action but also the partner's. This effect is particularly pronounced when the partners are close friends (Shafaei et al. 2020; Song et al. 2024), ingroup rather than outgroup (McClung et al. 2017), or individuals belief that the partner is behaving intentionally (Ruys and Aarts 2010; Sahaï et al. 2019; Tsai et al. 2008 but see Yamaguchi et al. 2019). Co‐representation is thus likely to support our intense cooperation, which has led to substantial interest in both the ontogenetic and phylogenetic origin of this mechanism.

Co‐representation can be operationalized with the joint Simon task (Sebanz et al. 2003). The original joint Simon task was built on a spatial compatibility task, where participants had to respond to one color with a right and the other with a left button press. Simultaneously, an irrelevant spatial stimulus was provided (i.e. a hand pointing to the right or left button), which could be compatible with the color stimulus or incompatible. The compatibility effect refers to longer reaction times and more mistakes in the incompatible trials when the hand points to the wrong button. In the half task, that is when a subject only can press one of the two buttons, this compatibility effect disappears. However, it reappears when the task is shared with a social partner who is responsible for the second button, even though for the subject the joint task remains identical to the half task. In the joint task, subjects thus not only represent their own task and action but also their partner's. This co‐representation has also been referred to as self‐other merging, and merging of perspectives, in the broader joint action literature (reviewed in Miss et al. 2022).

A particular focus has been whether co‐representation as measured with joint Simon tasks may be cognitively particularly demanding and unique to humans, which would explain why human cooperation stands out among primates. However, data from ontogenetic and phylogenetic perspectives are currently difficult to reconcile. In adult humans, co‐representation measured as joint Simon effects are often weak (e.g. Kiernan et al. 2012; Pfister et al. 2014; Sebanz et al. 2003; Yamaguchi et al. 2019) and reported to be stronger in people with stronger “perspective‐taking” abilities (Ruys and Aarts 2010 but see Iani et al. 2011). Moreover, it was found in 4–5 year‐old children, but not in 2–3‐year‐olds (Lin‐Lin et al. 2021; Milward et al. 2014; Saby et al. 2014). Task format apparently matters, because in a joint Simon paradigm that involved geometric figures and elicits a joint Simon effect in adults, Dudarev et al. 2021 failed to detect such an effect in 6–12‐year‐olds, whereas it was present in 6–8‐year‐olds (Iani and Rubichi 2025), and 13–18‐year‐olds (Shafaei et al. 2020) in a regular version of the task.

Together, this evidence from human participants is consistent with the idea that co‐representation is cognitively demanding and may require advanced cognitive abilities not in place before the age of 4–5 years. In particular, emerging Theory of Mind skills may be intricately linked to the requirement in co‐representation to shift the perspective from *self* to *other* and responsible for the contrasting results in 2–3 versus 4–5‐year‐olds, a period when Theory of Mind skills undergo significant development (Rakoczy 2022). Inhibitory control, on the other hand, is more likely to play a role in flexibly adjusting when to merge perspectives and engage in co‐representation, and when not, rather than for the emergence of co‐representation per se (Milward et al. 2017; Miss, Sadoughi, et al. 2022). This flexibility is important for cooperation success, because during cooperative interactions, periods where co‐representation is beneficial (e.g. when both participants have to perform the same action simultaneously) can alternate with periods when it is not (e.g. when two cooperation partners need to engage in complementary actions to achieve a joint goal). In the context of the joint Simon task, this flexibility is essential for cooperation success.

Whereas human developmental data is thus most consistent with co‐representation being a relatively late developing, cognitively demanding process, comparative evidence from nonhuman primates draws a different picture. Several nonhuman primate species have been tested with an auditory joint Simon task (Miss et al. 2022). In this task (see also  *Methods* below), two individuals next to each other work together to correctly respond to acoustic cues. These cues indicate that either a right‐ or a left‐hand response option has to be chosen, with each individual having access to, and thus being responsible for, only one of the two response options. To emphasize the jointness of the task, both individuals obtain a reward following a correct response. The acoustic cues are broadcast laterally, such that *compatible trials*, during which a cue indicating the response option on one side is played back from that same side, are easier than *incompatible trials*, during which the cue is played back from the opposite side, which induces a directional attention‐response conflict. This *incompatibility effect* is also referred to as Simon effect, operationalized with validated measures such as making more mistakes (i.e. manual response) and intention movements in the wrong direction (i.e. first heading directions) *in incompatible versus compatible trials* (Miss et al. 2022; Miss and Burkart 2018).

In the nonhuman primates, the overall response pattern is the same as in humans (in traditional, non‐acoustic versions of the joint Simon task: Sebanz et al. 2003 and subsequent studies, reviewed in Miss et al. 2022) and is as follows: The Simon effect is present equally when one individual is alone responsible for both response options (individual Simon effect, *full task*) and when two individuals are jointly engaged in the task with each of them being responsible for one half of the task (joint Simon effect, *joint task*). Importantly, the incompatibility effect disappears when no partner is present even though the individual is still responsible for only one response option (*half task*); it also disappears when a social partner is present but cannot engage with the task (*joint‐control task* which had been added in studies with nonhuman primates; Miss and Burkart 2018). This absence of a Simon effect in the half and the joint‐control task shows that the joint Simon effect is indeed elicited by a partner who is jointly engaged in the task. The overall response pattern has therefore been attributed to co‐representation or merging, that is either as task/action co‐representation where the individuals in the joint condition not only represent their own task and action but also their partner's (Sebanz et al. 2003), or as agent co‐representation where self‐other discrimination and conflict related to agent identification result in difficulties determining whose turn it is (Wenke et al. 2011; see Miss et al. 2022 for a detailed discussion).

When tested with this auditory joint Simon task, common marmoset monkeys (Miss and Burkart 2018), brown capuchin monkeys, Tonkean macaques (Miss et al. 2022), and also rats (Katsu and Okanoya 2024) show robust evidence for co‐representation, despite far less pronounced Theory of Mind and inhibitory control abilities compared to 4–5‐year‐old children (Krupenye and Call 2019; MacLean et al. 2014). Moreover, independently assessed inhibitory control was not correlated with individual variation in co‐representation or cooperation success in the nonhuman primates (Miss, Sadoughi, et al. 2022). Instead, across these tested species, the highest cooperation success was achieved by marmoset monkeys, who cooperate on a daily basis in their everyday lives because of their cooperative breeding social system where all group members help raising offspring. Marmosets were thus particularly skillful in flexibly adjusting when to co‐represent and when not, which helped them increase cooperation success. Intriguingly, they did not rely on inhibitory control for this because also within marmosets, cooperation success (which requires the flexible adjustment of co‐representation) was not correlated with independently assessed individual inhibitory control abilities (Miss, Sadoughi, et al. 2022). Instead, marmosets, but none of the other species, relied on mutual gaze: in joint, but not in joint‐control tasks, they were more likely to engage in mutual gaze in the period *after* the sound had been presented, and *before* any of the partners responded to the cue. Mutual gaze thus functioned as a behavioral coordination smoother in the cooperative marmosets, but not in the less cooperative macaques and capuchin monkeys (Miss et al. 2022). Overall, in contrast to the results from human children, the picture emerging from this set of results from nonhuman primates and rats thus rather suggests that co‐representation is a cognitively undemanding, fundamental default process, and overriding it can be achieved by behavioral coordination smoothers (mutual gaze).

**FIGURE 1:**
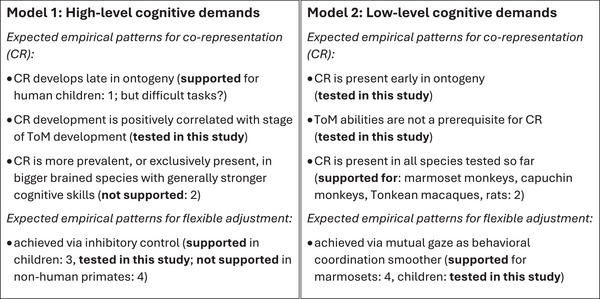
Co‐representation (CR) as cognitively demanding versus cognitively undemanding default process in ontogeny and phylogeny. Expected patterns in humans and across animals, together with available empirical evidence and predictions tested in the current study. References: 1: Lin‐Lin et al. 2021; Milward et al. 2014; Saby et al. 2014. 2: Katsu and Okanoya 2024; Miss et al. 2022; link between brain size and cognitive skills in nonhuman primates: Deaner et al. 2007; van Schaik et al. 2024. 3: Milward et al. 2017. 4: Miss, Sadoughi, et al. 2022; Miss and Burkart 2018.

Empirical data from human ontogeny so far thus predominantly support a cognitive high‐level model of co‐representation (Model 1 in Figure 1), where co‐representation increases at the age of steep progress in ToM understanding (around the age of 4 years), and where the flexibility to suppress it when needed is achieved with increasing inhibitory control. In contrast, comparative data from nonhuman species are more supportive for a cognitive low‐level model (Model 2 in Figure 1), where co‐representation is a default that does not require any ToM abilities and the flexibility to suppress it is achieved via mutual gaze as a coordination smoother. Moreover, neither co‐representation, nor its flexible adjustment, were correlated with brain size across species, which is an important predictor for cognitive abilities among nonhuman primates (Deaner et al. 2007; van Schaik et al. 2024), and is smaller in marmosets compared to the other four species, smaller in capuchins compared to macaques and humans, and smaller in macaques compared to humans.

However, to date it cannot be excluded that the relatively late onset of co‐representation during human ontogeny may be due to cognitive task demands other than co‐representation (in particular task formats, verbal instructions, and response formats: reviewed in Miss et al. 2022). It is therefore crucial to obtain directly comparable data to allow for direct, formal comparisons between species. We tested 2–4‐year‐old human children with the nonhuman primate version of the joint Simon task (Miss et al. 2022) which is optimized to test nonverbal subjects with limited memory spans and poses no linguistic demands. In parallel, we assessed the children's stage of Theory of Mind development and inhibitory control abilities which allowed us to address (i) whether co‐representation indeed only emerges in the oldest age group, (ii) how it is related to individual differences in Theory of Mind developmental stage and inhibitory control, (iii) what mechanisms children use to override co‐representation to improve cooperation success (mutual gaze as behavioral coordination smoother, communication, and/or inhibitory control), and (iv) how their performance compares to other species.

If co‐representation is indeed a cognitively undemanding, early default mode of processing joint action (i.e. the low‐level model in Figure 1) we predicted an early onset and a negative effect of age on co‐representation, as well as no positive correlation with the stage of Theory of Mind development. If flexible adjustment of co‐representation to increase cooperation success is achieved in cognitively undemanding ways, children may rely on mutual gaze as a cognitively undemanding coordination smoother as marmosets. Nevertheless, in particular older children may in addition also rely on more cognitive mechanisms, such as verbal communication and increasing inhibitory control. This would be manifest in a negative correlation between inhibitory control and co‐representation.

Across species, cooperation success in the joint Simon task, mediated by the ability to flexibly switch between merging in co‐representation or not, was highest in the species which routinely cooperates in their everyday lives, the common marmosets compared to the less cooperative capuchin monkeys and Tonkean macaques (Miss et al. 2022; Miss, Sadoughi, et al. 2022). We expected that humans would follow the same trend as the other primate species. Since humans engage even more in routine cooperation (Burkart et al. 2009) than marmosets, we predicted highest cooperation success in human children.

## 2 Methods

### 2.1 Participants

#### 2.1.1 Human children

Participants were 38 children between 2.0 and 4.2 years of age (mean age ± SD = 39.50 ± 6.96 months; range = 24–51 months; 11 female) from three day care centers in urban or suburban areas of Zurich, Switzerland. Participants’ descent was of White or mixed‐ethnic groups with families of medium to high socioeconomic status. Their language preferences were (Swiss‐) German or English. Eight participants either left the day‐care center before the end of the study or were unwilling to participate and were thus excluded from analyses. All remaining 30 participants carried out the four Simon task conditions and subsequently the ToM and inhibition test battery. The study was approved by the Ethics Committee of the Faculty of Arts and Social Sciences of the University of Zurich, Switzerland, and written informed consent was obtained for all participants from a parent or legal guardian. Testing took place in separate rooms of the day care centers.

#### 2.1.2 Data from nonhuman primates

For the interspecific analyses, we used the data from Miss and Burkart 2018, as well as Miss et al. 2022. This data set includes 10 common marmosets (5 females), 7 capuchin monkeys (3 females), and 7 Tonkean macaques (2 females). Common marmosets are, like humans, and cooperative breeders that cooperate intensely on a daily basis (Burkart et al. 2009; Snowdon and Cronin 2007). Capuchins, Tonkean macacques (Ciani et al. 2012; de Waal 2000; Thierry et al. 1994; Thierry 2007; Weaver and de Waal 2003) and in particular marmosets (Schaffner and Caine 2000) show high levels of social tolerance, whereas extensive allomaternal care and cooperation during everyday interactions with group members is exclusively present in the marmoset monkeys (Burkart et al. 2014; Mendres and de Waal 2000; Perry and Rose 1994; Petit et al. 1992; Thierry et al. 1994). General cooperativeness is thus highest in humans, followed by marmosets, capuchin monkeys, and Tonkean macaques. In contrast, overall brain size, which is a better predictor for cognitive abilities among nonhuman primates than brain size relative to body size (Deaner et al. 2007; van Schaik et al. 2024), is smallest in marmosets, and smaller in capuchins compared to macaques and humans, and smaller in macaques compared to humans.

### 2.2 The Auditory Version of the Joint Simon Task

**FIGURE 2:**
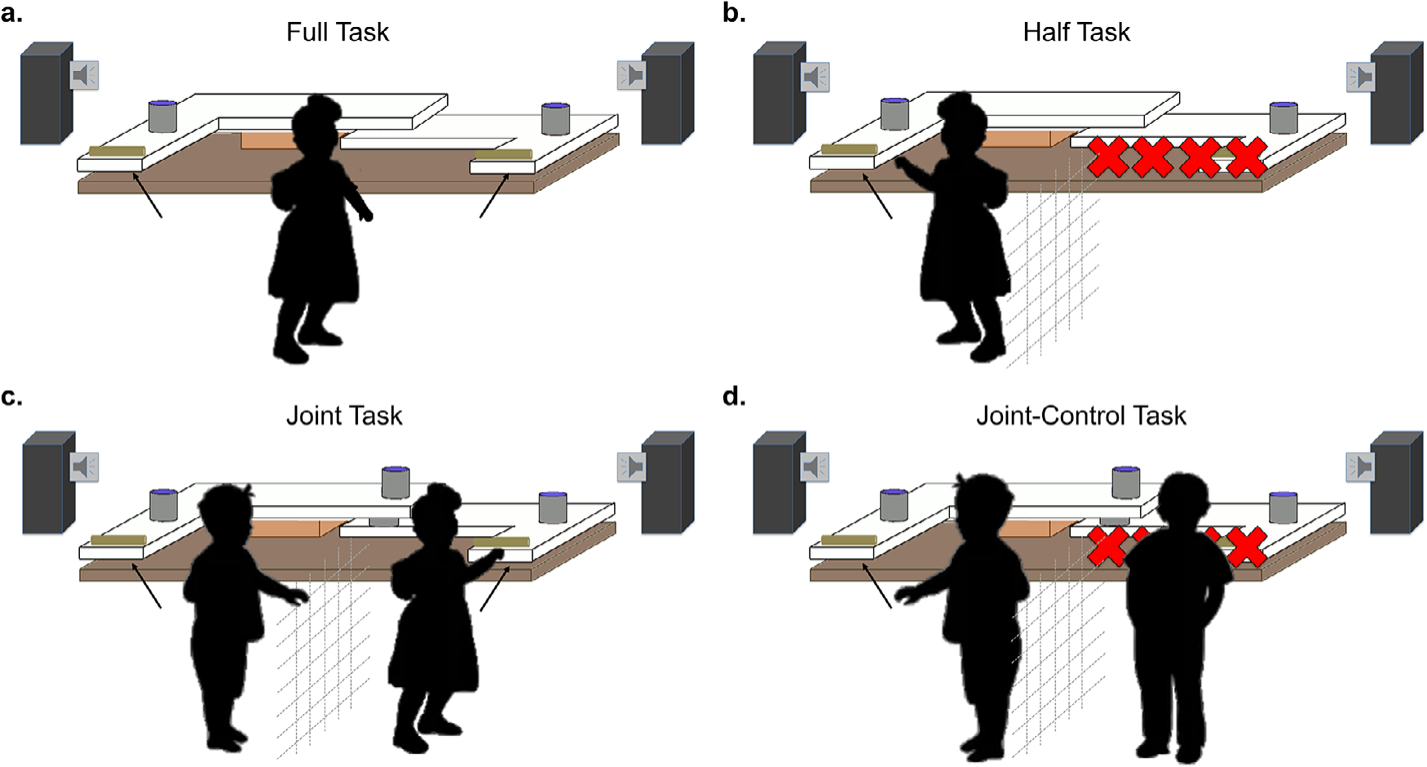
Testing device and experimental design. Setup in the four task conditions (a) full task, (b) half task, (c) joint task, and (d) joint‐control task. In every trial, either sound “L” or “R” was broadcast from one of the two lateral speakers and demanded pulling either the left‐hand drawer (sound “L”) or the right‐hand drawer (sound “R”). If the correct drawer was pulled, a reward could be retrieved from the grey cup(s). In the joint task, the outer cup became available to the active puller and the middle cup to the partner child; both partners were thus rewarded for a correct choice in the joint task, but only one in the other three conditions.

***Setup and apparatus***. The children were tested with an auditory version of the Simon task previously used with monkeys (Figure 2). The setup consisted of a playpen for the participants, and an apparatus in front of the playpen that could be revealed by opening a curtain and the participants could reach out of the playpen to pull the handle of the apparatus (see, Figure S1). The apparatus could be positioned in front of the playpen with a distance appropriate to the child's body size and arm length. Two sliding drawers, each with a handle to grab, were attached to the apparatus with a mechanism allowing only one exclusive choice per trial (see, Figure S1). A cord connected the back sides of the two drawers, which resulted in a backwards and out of reach movement of the second drawer for the other child as soon as one drawer was pulled. In case the correct drawer was pulled (according to the sound), the participants could open a cup and retrieve a small reward (Figure 2). In the joint task, two cups fixed onto each drawer enabled the retrieval of a reward for both, the active puller and the partner child. In the joint‐control task, two cups were fixed onto each drawer as in the joint task, but the access to the drawer was blocked for the partner child, and therefore the second cup remained out of reach. As rewards, we used a random mix of stamps and stickers, which the children could use to create their own card, different little toy objects, Lego bricks, puzzle pieces, and occasionally a sweet or a salty snack. A partition grid rendering possible visual and auditory contact between task partners was used to divide the playpen into two equally sized compartments in the half, joint, and joint‐control task conditions. A curtain attached to a wooden frame covering the length of the playpen was used to bait the cups out of sight of the children. A cross on the floor within the playpen and stickers showing a smiling face attached to the curtain in front of the children were used to make starting positions and attendance as consistent as possible before the start of a trial (see, Figure S1). The auditory stimuli (sound “L” and “R”) consisted of the identical two piano tone sequences previously used with the monkeys.

***Task conditions***. The subjects first learned to discriminate between two sounds, each indicating that a reward would become available if the correct answer option was chosen (i.e. pulling the left one of two drawers if sound “L” was played back, and the right one if sound “R” was played back). Once this discrimination was mastered (see section 1.1. for details), a full, a half, a joint, and a joint‐control condition were run. For the full task (Figure 2a), the sounds were played back from either the right‐ or left‐hand side, resulting in incompatible (stimulus configurations with a mismatch between sound and correct response side) and compatible trials (individual Simon effect expected). For the half task (Figure 2b), the access to the second drawer was blocked, but the sounds were still played back laterally (individual Simon effect expected to disappear). In the joint task (Figure 2c), the situation for the participant was identical as in the half task but a partner was added who could solve the other half of the task (joint Simon effect expected). In the joint‐control condition finally (Figure 2d), the situation was identical for both participants as in the joint condition, with the exception that the partner could not access the drawer (joint Simon effect expected to disappear). This joint‐control condition was used to exclude low‐level explanations of the joint Simon effect, such as spatial response coding (Dittrich et al. 2012; Dolk et al. 2014; Miss and Burkart 2018; Yamaguchi et al. 2019). To qualify as co‐representing, participants had to show a Simon effect (individual and joint) in the full and the joint task condition but must not show a Simon effect in the half and the joint‐control condition.

***Procedures***. At the beginning of a trial, participants stayed within a playpen to standardize starting positions and moving options. In every trial, one of the two sounds was broadcast from one of the two lateral speakers. Sound “L” demanded pulling the handle on the left‐hand side whereas sound “R” demanded pulling the drawer on the right‐hand side. If the correct handle was pulled, the grey reward container now into reach could be flipped over to retrieve the reward. Each of the four task conditions contained compatible (i.e. “L” broadcast from the left‐hand side and “R” from the right‐hand side) and incompatible trials (i.e. “L” broadcast from the right‐hand side and “R” from the left‐hand side).

Each participant completed four test sessions in the four task conditions in a pseudo‐randomized order, resulting in 16 sessions in total per participant. In the social task conditions (i.e. the joint and the joint‐control task), participants were chosen at random for the formation of pairs. Per day, only one task condition and session were administered resulting in a total test duration of 20–40 min. The test session consisted of 12 trials with breaks in between if needed. The order of the sound stimuli and the sides they were broadcast, as well as the side position of the participants were pseudo‐randomized and counterbalanced with the restriction of not choosing a particular sound or a particular side more than twice in a row. For more details of the procedures, see section 1.1.

### 2.3 ToM and Inhibition Tasks

In addition, we administered a set of five ToM tests designed and validated to assess young children's stage of Theory of Mind understanding (Kristen et al. 2006; Wellman and Liu 2004). Importantly, these tests represent ascending levels of Theory of Mind understanding because they were designed to fit a Guttman scale, indicating that participants solving a more difficult test can also solve all tests that are classified less difficult. Empirical tests show this is the case also in small samples (*n* = 30) of children in the age range tested here (Burkart and Rueth 2013) and the overall ranking provides a reliable measure of the stage of Theory of Mind understanding. The tests were presented according to their ascending level of difficulty (i.e. diverse desire test, diverse belief test, knowledge access test, explicit false belief test, and contents false belief test; Figure S2).

We also measured motor inhibitory control, with an adapted version of the day‐night Stroop task (Gerstadt et al. 1994; Milward et al. 2017) and the statue task (Korkman et al. 1998, NEPSY statue task). In the day‐night Stroop task, the children were instructed to touch an image depicting the moon and stars when the experimenter said “day”, and to touch an image depicting the sun when the experimenter said “night”. In the statue task, the child was asked to maintain a fixed body position with eyes closed during 75 s and to inhibit the impulse to respond to sound distractors, Figure S3. For the details of task administration see section 1.2.

### 2.4 Data Coding

***The Simon task***. Interference effects can be detected as mistakes in manual response choices, in first heading directions (intention movements), or reaction times. Nonhuman primates showed co‐representation in mistakes and first heading directions but not in reaction times (Miss et al. 2022; Miss and Burkart 2018). Mistakes are easy to code but face the problem that in half and joint‐control conditions, they are possible in only 50% of all cases because the access to the second response option is blocked. Since nonhuman primates, and presumably also young children, tend to always respond in a given trial, this response variable can be problematic (Martínez et al. 2024). This problem can be avoided when instead focusing on first heading directions, or intention movements, that is the first movement toward one of the two targets immediately after the stimulus presentation which can occur in all trials. The first heading direction is thus the more reliable and also more sensitive measure because it can also capture only slight action tendencies that eventually are stopped and don't lead to a pull (i.e. a manual response choice). Finally, interference effects in adult humans are often reported with reaction times as outcome variable. While able to capture very subtle differences, they are problematic with pre‐ or nonverbal subjects because one cannot explain that they are supposed to respond as fast as possible. Using reaction times with pre‐ and nonverbal subjects would thus require extensive training. In the present study, to be able to directly compare to the nonhuman primate data, we used first heading directions as primary outcome variable as well as manual response choices.

The participants’ manual response choices (i.e. correct or incorrect response choice resulting either in a reward or not) were recorded directly on paper and all sessions were video recorded (with a perspective from the top and the front) to verify the records and quantify additional behavioral response variables with the software INTERACT (Mangold GmbH, version 18.5.5.1). A trial was coded from the onset of the stimulus until the participant firmly grabbed the handle (thus prior to the start of the pulling movement and thus prior to responding to the stimulus) or, in case of the half and the joint‐control task, until the participant firmly grabbed the handle of the accessible side or pointed at or made a verbal reference to the inaccessible side. As in the nonhuman primates, we also coded for each trial the participant's first heading direction (i.e. the first subtle body orientation or movement toward one response side), the gazing behavior directed at the partner (in the joint and joint‐control task) and the communication behavior (gestural and verbal expressions in the joint and the joint‐control task, see section 1.3 for more details on behavioral definitions).

We assessed interrater reliability separately for all the response variables by analyzing 13% of the recorded sessions of all tasks by a second rater. We thus analyzed interrater agreement for *manual response choices* (Cohen's Kappa (*Κ*) = 1), *first heading directions* (*K* = 0.95, 95% CI [0.93, 0.98]), *mutual gaze* (*K* = 0.9, 95% CI [0.81, 0.98]), and all communication categories. The latter included *ask* (*K* = 0.97, 95% CI [0.92, 1]), *exclaim* (*K* = 0.86, 95% CI [0.58, 1]), *inform* (*K* = 0.93, 95% CI [0.89, 0.97]), and *correct* (*K* = 0.91, 95% CI [0.81, 1]).

***ToM and inhibition tasks***. The participants’ answers were recorded directly on paper where applicable and all sessions were video‐recorded to check the paper records and to complete the data coding by the use of the software INTERACT (Mangold GmbH, version 18.5.5.1). A correct response in each of the five ToM tests (Kristen et al. 2006; Wellman and Liu 2004) was rewarded with one point, resulting in a total ToM score per participant ranging from 0 to 5. For the tests No. 3, 4, and 5 a point was given only if both the target and the control question were answered correctly.

For the day‐night Stroop task (Gerstadt et al. 1994; Milward et al. 2017), we measured accuracy by coding each answer (i.e. correct or incorrect first touch or first point in case the participant consistently did not touch the image), resulting in a total score ranging from 0 to 12.

For the statue task, we defined errors according to (Korkman et al. 1998, NEPSY statue task, see section 1.3). The total score in the statue task could range from 0 to 30, with higher scores indicating higher inhibitory control.

We assessed interrater reliability by analyzing 13% of the recorded sessions (randomly selected participants per task) by a second rater, resulting in high interrater agreement for the scores in the *ToM tests* (*K* = 1), the scores in the *day‐night Stroop task* (*K* = 1), and the scores in the *statue task* (*K* = 0.92, 95% CI [0.80, 1]).

### 2.5 Data Analysis

All statistical analyses were performed in R (version 3.5.3). We used generalized linear mixed‐effects models (glmms; function “glmer,” package “lme4”; Bates et al. 2015). The full model with all fixed effects of interest was always compared to the null model only including the intercept and random effects (or to a control model in case of the analyses of cognitive factors only, see below) using likelihood ratio tests (function “Anova,” package “car”; Fox and Weisberg 2019). Variance Inflation Factors (VIFs) were calculated to examine if predictors did not violate any multicollinearity assumptions using the package “car” (Fox and Weisberg 2019; all VIF scores < 3). The proportion of the total variance accounted for by the models was assessed by the conditional *R* ^2^_GLMM_ values using the package “MuMIn” (Bartoń 2025). All figures were generated using the package “ggplot2” (Wickham 2016).

***Emergence of co‐representation during ontogeny***. To assess whether the children showed an individual and a joint Simon effect indicative of co‐representation, we calculated two binomial glmms with either the children's manual response choice (correct or incorrect) or first heading direction (toward the correct or incorrect response side) as dependent variable.

We were interested in the effects of compatibility (compatible and incompatible trials), task (full, half, joint, and joint‐control task) and age, and their two‐ and three‐way interactions in both models. If children showed an individual and a joint Simon effect in any of the dependent variables, we would expect an interaction effect between task and compatibility. We further included sex, session number, response side, and sound stimulus as fixed factors in the full models to test for potential learning effects over time, sex differences, and preferences for one of the two response sides or sounds. For the fixed factor *task*, we set *a* *priori* contrasts to compare the experimental with the control conditions (i.e. full and joint task vs. half and joint‐control task) and to compare within each set of task conditions (i.e. full vs. joint task and half vs. joint‐control task). For the fixed factor *session* number, we set *a* *priori* polynomial contrast to test for trends across time. Session number, and dyad ID (specifying the individual in the solo task conditions or the dyad in the social task conditions) nested in childcare group were included as random factors in both models.

***Correlation with ToM and IC***. We first tested whether in our sample, we would find the expected developmental progression in the childrens’ ToM understanding and inhibitory control with Spearman rank correlation analyses (function “cor.test”, package “stats”; R Core Team 2019) between both ToM abilities (ToM tasks score) and inhibitory control abilities (day‐night Stroop task score and statue task score), and age.

To analyze if ToM and inhibitory control skills predicted the strength of interference effects (the joint Simon effect—i.e. co‐representation) as assessed by the first heading direction, we calculated binomial glmms on the data in each task condition and examined the effects of ToM scores and composite inhibitory control scores (see section 2.2) on the interference effects. The models included session number, and dyad ID (specifying the individual in the solo task conditions or the dyad in the social task conditions) nested in childcare group as random factors and were compared to a control model containing only the control factor (compatibility) and the random factors.

***Mutual gaze and communicative cues***. First, we combined all verbal and gestural expressions that were directly related to the response selection problem and determination of turn responsibility into a binary communication variable. This variable therefore contained the information whether an expression belonging to one of four categories (i.e. ask, exclaim, inform, and correct) had occurred in a given trial. In the social test conditions (joint and joint‐control task), to assess whether and how visual monitoring of the partner and the expression of communicative cues changed according to the task condition, we calculated two binomial glmms with either mutual gaze (occurred or not) or communicative cues (occurred or not) as dependent variable. In both models, we examined the effects of task, compatibility and age. We included session number, and dyad ID nested in childcare group as random factors in both models.

***Comparisons between species***. To compare co‐representation between species, we first calculated a binomial glmm on the response choices in the joint task and examined the effect of species to test whether the four groups (humans, marmoset monkeys, capuchin monkeys, and Tonkean macaques) differed in their cooperation success (i.e. correct trials in the joint condition). Next, we analyzed whether the tested groups differed in the strength of their individual and joint Simon effect measured with their first heading direction as the most sensitive variable. We calculated a binomial glmm and examined the effects of compatibility, task, and species, and their interactions on the first heading directions. In both models, contrasts were set *a* *priori* for the fixed factor species to compare the effects between the cooperatively breeding species (humans and common marmosets) and the independently breeding ones (capuchin monkeys and Tonkean macaques), and to compare within each pair of species (humans vs. marmoset monkeys, and capuchin monkeys vs. macaques). We included session number, and dyad ID (specifying the individual in the solo task conditions or the dyad in the social task conditions) nested in species as random factors.

Further, we tested whether the four species differed in their visual monitoring behavior in the social test conditions (i.e. joint and joint‐control conditions). We calculated a binomial glmm with mutual gaze as dependent variable and examined the effects of task, species, and their interaction. Contrasts were set *a* *priori* to compare mutual gaze between the cooperatively breeding species (humans and common marmosets) and the independently breeding ones (capuchin monkeys and Tonkean macaques), and to compare within each pair of species. We included session number, and dyad ID nested in species as random factors.

## 3 Results

**FIGURE 3:**
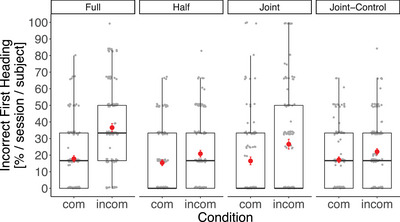
Children's incorrect first heading directions in the four task conditions, calculated per session, and individual. The Simon effect reflects the difference in the percentage of first movements toward the incorrect response side between compatible (com) and incompatible (incom) trials, which is significantly larger in the experimental conditions (full and joint task) than in the control conditions (half and joint‐control task). The boxes and whiskers represent medians and lower and upper quartile scores. Error bars (in red) represent standard errors of the mean.

***Emergence of co‐representation during ontogeny***. First, we examined the presence of a Simon effect in the full and the joint, but not the half and the joint‐control condition, as is indicative of co‐representation. We therefore analyzed the children's accuracy in the first heading direction, that is the first subtle body orientation or movement toward either the correct or incorrect response side, as well as the accuracy in the response choice (correct or incorrect manual choice). An individual and a joint Simon effect was observable in the participants’ first heading direction. The full model explained significantly more variation than the null model [*χ* ^2^_14_ = 196.48, *p* < 0.001, pseudo‐*R* ^2^ *_c_* = 0.23, *∆*AIC = 168.5, *N*_total_ = 4979, *N*_individuals_ = 30] and revealed a significant effect of compatibility, age, session and sex, and a significant two‐way interaction between task and compatibility (Table S1). While first headings toward the incorrect response side generally occurred more often in incompatible than compatible trials, this difference was significantly larger for the experimental conditions (full and joint task) compared with the control conditions (half and joint‐control task), *β* ± SE = 0.29 ± 0.08, 95% CI [0.13, 0.44], *z* = 3.70, *p* < 0.001 (Figure 3).

**FIGURE 4:**
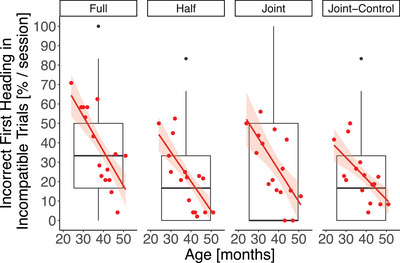
Children's incorrect first heading directions (mean percentages calculated per session) in incompatible trials of the four task conditions according to their age. Each point indicates a mean value per age. The boxes and whiskers represent medians and lower and upper quartile scores. The regression lines show the correlations between incorrect first heading directions and the children's age. The shaded areas display 95% confidence intervals.

Incorrect first headings occurred more often in younger participants than older ones, *β* ± SE = −0.08 ± 0.01, 95% CI [−0.10, −0.05], *z* = ‐6.63, *p* <  .001, indicating stronger interference effects in younger children than older ones in all task conditions (Figure S4). This age effect was particularly strong in incorrect heading directions in incompatible trials (Figure 4). Finally, boys showed slightly stronger interference effects than girls, *β* ± SE = 0.34 ± 0.16, 95% CI [0.02, 0.65], *z* = 2.09, *p* =  .037, and the linear trend of session (*β* ± SE = −0.22 ± 0.08, 95% CI [−0.37, −0.07], *z* = ‐2.90, *p* =  .004) revealed a general learning effect over time. Incorrect first headings occurred more often in the first than in the last session of the full, the joint and the joint‐control task (*full task* mean percentage 29.58 ± SE 3.63 versus 24.91 ± SE 3.72; *half task* 18.61 ± SE 3.34 versus 19.34 ± SE 3.30; *joint task* 21.20 ± SE 4.08 versus 19.23 ± SE 3.45; *joint‐control task* 25.08 ± SE 2.83 versus 14.42 ± SE 2.68).

An individual and a joint Simon effect was not observable in the participants’ manual response choices (actual pulls), as incorrect response choices were rare and did not differ in incompatible and compatible trials between the experimental conditions (full and joint task) and the control conditions (half and joint‐control task; section 2.1, Figure S5 and S6). Overall, the childrens’ error rate for response choices was low in both the experimental tasks (mean percentage *full task* 12.71 ± SE 2.85; *joint task* 11.92 ± SE 2.58) and the control tasks (mean percentage *half task* 14.18 ± SE 2.73; *joint‐control task* 15.66 ± SE 3.07). The low error rate in the joint task corresponds to the childrens very high cooperation success (see comparison between species below).

In sum, trial compatibility had an effect on first headings and this effect changed magnitude according to the task conditions: incompatible trials showed a higher proportion of incorrect first headings than compatible trials and this difference was larger in the full and the joint task than in the half and the joint‐control task, as is indicative of an individual and joint Simon effect, that is co‐representation. This interference effect, and thus co‐representation, was stronger in younger children than older ones. Overall, co‐representation was weak and detectable only in first heading directions but not in choices, which resulted in a high overall cooperation success in the joint task.

***Correlation between Theory of Mind, IC and co‐representation***. The children's performance in the ToM tasks, the day‐night Stroop task and the statue increased with age, validating that the sample size was large enough to detect these expected age effects (section 2.2; Figure S7). We thus analyzed the influence of ToM and inhibitory control on the strength of co‐representation (i.e. interference effects measured with the first heading direction).

The full model including compatibility, ToM scores and composite inhibitory control scores explained co‐representation in the joint task significantly better than the control model containing compatibility as control factor [*χ* ^2^_2_ = 22.93, *p* < 0.001, pseudo‐*R* ^2^ *_c_* = 0.37, *∆*AIC = 18.93, *N*_total_ = 713, *N*_individuals_ = 30]. It showed a significantly negative effect of inhibition (*β* ± SE = −0.06 ± 0.02, 95% CI [−0.10, −0.03], *z* = ‐3.74, *p* < 0.001), but no effect of ToM (*β* ± SE = ‐0.02 ± 0.21, 95% CI [−0.42, 0.39], *z* = −0.09, *p* =  .93). The full models also explained interference effects in the other task conditions significantly better than the control models [*full task*: *χ* ^2^_2_ = 13.58, *p* = 0.001, pseudo‐*R* ^2^ *_c_* = 0.24, *∆*AIC = 9.5, *N*_total_ = 1416, *N*_individuals_ = 30; *joint‐control task*: *χ* ^2^_2_ = 11.26, *p* = 0.004, pseudo‐*R* ^2^ *_c_* = 0.19, *∆*AIC = 7.20, *N*_total_ = 1418, *N*_individuals_ = 30; *half task*: *χ* ^2^_2_ = 14.57, *p* < 0.001, pseudo‐*R* ^2^ *_c_* = 0.26, *∆*AIC = 10.50, *N*_total_ = 1432, *N*_individuals_ = 30]. They all showed a significantly negative effect of inhibition (*full task*: *β* ± SE = ‐0.04 ± 0.02, 95% CI [−0.07, −0.001], *z* = −2.03, *p* = 0.042; *joint‐control task*: *β* ± SE = −0.03 ± 0.01, 95% CI [−0.05, −0.01], *z* = −2.79, *p* = 0.005; *half task*: *β* ± SE = −0.05 ± 0.02, 95% CI [−0.09, −0.02], *z* = −3.17, *p* = 0.002) but not ToM. Thus, stronger inhibitory control ability was associated with weaker co‐representation, and further with weaker interference effects in the full, half, and joint‐control task.

**FIGURE 5:**
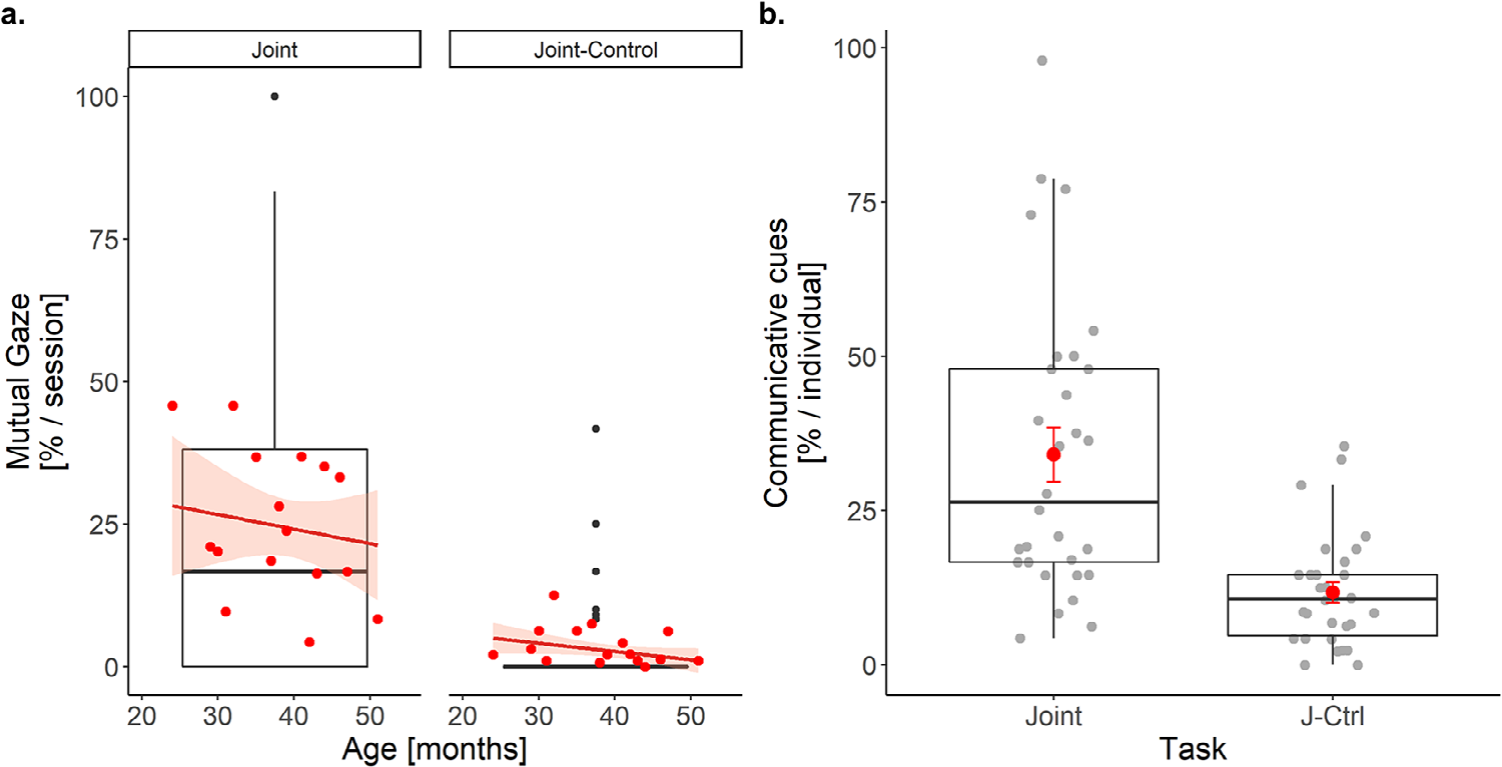
Children's mutual gaze and communicative expressions in the social test conditions. Mean percentages of (a) mutual gaze calculated per session according to the children's age (each point indicating a mean value per age), and (b) communicative (verbal or gestural) cues directly related to the response selection problem and determination of turn responsibility (i.e. belonging to either of the categories “ask”, “exclaim,” “inform,” “correct”) calculated per individual (a jitter function was applied to make overlapping data points more visible). The boxes and whiskers represent medians and lower and upper quartile scores. Error bars (in red) represent standard errors of the mean. The regression lines show the correlations between mutual gaze and the children's age. The shaded areas display 95% confidence intervals.

***Mutual gaze and communicative cues***. Mutual gaze is a social cue that potentially helps to suppress co‐representation and thus increase cooperation success in the joint task (Miss and Burkart 2018). We therefore analyzed the children's mutual gaze behavior in the social test conditions (joint and joint‐control task). The full model including task, compatibility, and age explained mutual gaze significantly better than the null model [*χ* ^2^_3_ = 197.07, *p* < 0.001, pseudo‐*R* ^2^ *_c_* = 0.49, *∆*AIC = 191.1, *N*_total_ = 2131, *N*_individuals_ = 30], and revealed no effect of compatibility and a trend for age. The children engaged more often in mutual gaze in the joint than the joint‐control task, *β* ± SE = −2.84 ± 0.24, 95% CI [−3.30, −2.37], *z* = −11.97, *p* < 0.001, and younger children tended to engage in mutual gaze more often than older ones (*β* ± SE = −0.04 ± 0.02, 95% CI [−0.08, 0.002], *z* = −1.85, *p* = 0.065; Figure 5a).

We also examined communicative cues, categorized as either a verbal or a gestural expression directly referring to the response selection problem and determination of turn responsibility in the social task conditions. The full model including task, compatibility, and age explained communicative expressions significantly better than the null model [*χ* ^2^_3_ = 292.41, *p* < 0.001, pseudo‐*R* ^2^ *_c_* = 0.45, *∆*AIC = 286.4, *N*_total_ = 2131, *N*_individuals_ = 30]. Communicative expressions occurred more often in the joint than the joint‐control task, *β* ± SE = −2.39 ± 0.15, 95% CI [−2.68, −2.11], *z* = −16.44, *p* < 0.001 (Figure 5b). Neither compatibility nor age showed a significant effect.

In the joint task, communicative cues were correlated with mutual gaze within the same trial (*β* ± SE = 1.47 ± 0.26, 95% CI [0.97, 1.97], *z* = 5.71, *p* < 0.001; Figure S8). Adding mutual gaze as a predictor variable significantly improved the model fit [*χ* ^2^_1_ = 34.88, *p* < 0.001, pseudo‐*R* ^2^ *_c_* = 0.45, *∆*AIC = 32.88, *N*_total_ = 713, *N*_individuals_ = 30]. The effect persisted when analyzing the trials of the joint and the joint‐control task together (section 2.3).

**FIGURE 6:**
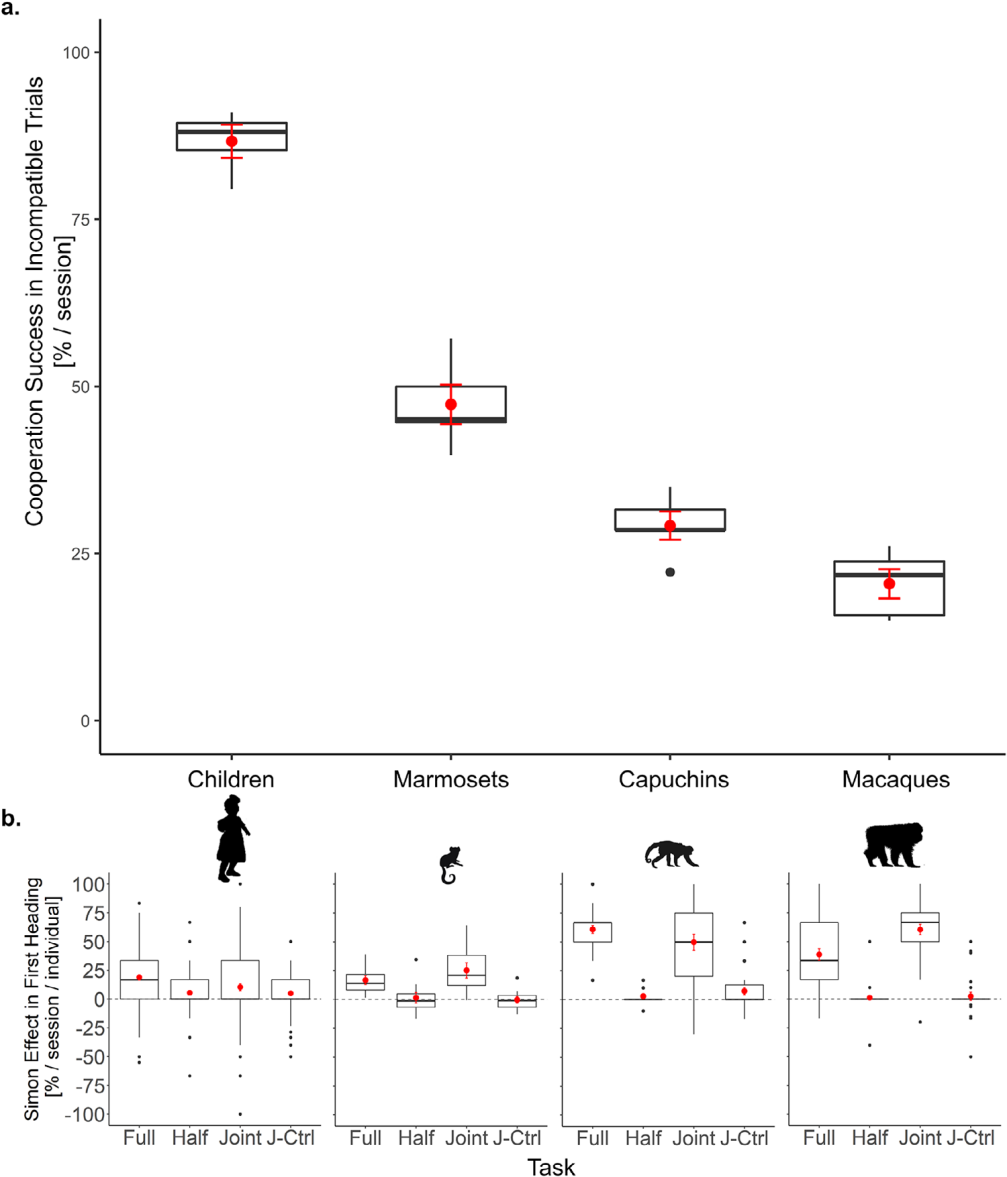
(a) Cooperation success in the dyads of the four tested species in incompatible trials of the joint task. Cooperation success was calculated as observed correct response choices resulting in a retrievable reward for both partners in incompatible trials per session and species. The boxes and whiskers represent medians and lower and upper quartile scores. Error bars (in red) represent standard errors of the mean. (b) Simon effect in first heading directions in the four task conditions. The Simon effect is shown as the difference in the percentage of first movements toward the incorrect response side between incompatible and compatible trials calculated per session and individual in the children, the marmoset monkeys, the capuchin monkeys, and the Tonkean macaques. The boxes and whiskers represent medians and lower and upper quartile scores. Error bars (in red) represent standard errors of the mean.

***Comparisons between species***. The cooperation success in the joint task differed significantly between the four tested species [*χ* ^2^_3_ = 15.50, *p* = 0.001, pseudo‐*R* ^2^ *_c_* = 0.42, *∆*AIC = 9.5, *N*_total_ = 1750, *N*_individuals_ = 53]. The children and the marmoset monkeys made more correct choices resulting in a reward for both partners, than the capuchin monkeys and the Tonkean macaques, *β* ± SE = −0.71 ± 0.19, 95% CI [−1.07, −0.34], *z* = ‐3.79, *p* < 0.001. The children also significantly differed from the marmoset monkeys in their cooperation success, *β* ± SE = −1.18 ± 0.26, 95% CI [−1.69, −0.68], *z* = −4.62, *p* < 0.001; Figure S9). The pattern was particularly strong in incompatible trials (Figure 6a), together indicating stronger co‐representation in Tonkean macaques and capuchin monkeys than marmosets and children.

We next examined whether the four tested groups differed in the strength of their individual and joint Simon effect measured with their first heading direction as the most sensitive behavioral variable in this task design. The full model including compatibility, task, species, and their two‐ and three‐way interaction explained significantly more variability in the first heading direction than the null model [*χ* ^2^_31_ = 565.52, *p* < 0.001, pseudo‐*R* ^2^ *_c_* = 0.24, *∆*AIC = 504, *N*_total_ = 10235, *N*_individuals_ = 54]. First, the individual and the joint Simon effect were stronger in the less cooperative, independently breeding species (capuchin monkeys and Tonkean macaques) than in the cooperatively breeding ones (humans and marmoset monkeys), *β* ± SE = 0.34 ± 0.05, 95% CI [0.24, 0.45], *z* = 6.54, *p* < 0.001. Second, the Tonkean macaques, contrary to the capuchin monkeys, showed a stronger joint than individual Simon effect, *β* ± SE = ‐0.53 ± 0.14, 95% CI [−0.82, −0.25], *z* = −3.70, *p* < 0.001. The marmoset monkeys as well, contrary to the children, showed a stronger joint than individual Simon effect, *β* ± SE = −0.18 ± 0.08, 95% CI [−0.34, −0.02], *z* = −2.20, *p* = 0.028 (Figure 6b; Table S2).

**FIGURE 7:**
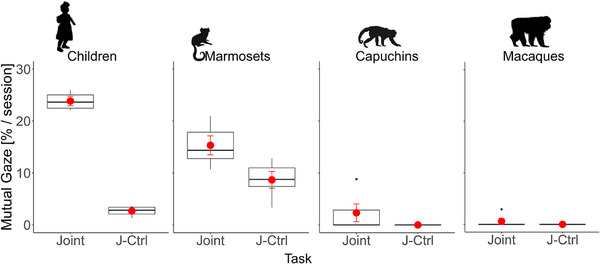
Mutual gaze between partners in the social test conditions. Mean percentages calculated per session of mutual gaze behavior in the joint and the joint‐control task in the children, the marmoset monkeys, the capuchin monkeys, and the Tonkean macaques. The boxes and whiskers represent medians and lower and upper quartile scores. Error bars (in red) represent standard errors of the mean.

We finally investigated whether the four tested species differed in their mutual gaze behavior in the social test conditions. The full model including task and species, and their two‐way interaction explained significantly more variability in mutual gaze behavior than the null model [*χ* ^2^_4_ = 178.41, *p* < 0.001, pseudo‐*R* ^2^ *_c_* = 0.55, *∆*AIC = 170.4, *N*_total_ = 4291, *N*_individuals_ = 53] (Table S3). First, mutual gaze was overall far less prevalent in the independently breeding species (capuchins and Tonkean macaques) than in the cooperatively breeding ones (humans and marmosets; *β* ± SE = −1.78 ± 0.31, 95% CI [−2.39, −1.16], *z* = ‐5.64, *p* < 0.001). Second, if mutual gaze was present, it occurred more often in the joint than the joint‐control task in all tested groups, *β* ± SE = −1.76 ± 0.15, 95% CI [−2.06, −1.47], *z* = −11.90, *p* < 0.001 (Figure 7).

## 4 Discussion

Our results support the cognitive low‐level model of co‐representation (Figure 1). Co‐representation was already present in the youngest children and decreased with age, and individual levels of ToM understanding were not correlated with co‐representation. Over the period of 2–4 years, the young children thus got increasingly better at *avoiding* the detrimental effect of pre‐existing co‐representation, and so could increase cooperation success. Finally, compared to the other primate species, human co‐representation was weaker, even in the two‐year‐olds.

The presence of co‐representation already in two‐year‐old children contrasts with previous reports who found it only in older children (only in 4–5 but not in 2–3 year‐olds: Lin‐Lin et al. 2021; Milward et al. 2014; Saby et al. 2014; only in adults but not in 6–12‐year‐olds: Dudarev et al. 2021). This contrast, we argue, is the result of the low‐cognitive task demands of the auditory version of the joint Simon task, which was deliberately designed to minimize such demands (Miss and Burkart 2018). The most important contrast is that the auditory version includes an auditory cue dimension (the sounds that indicate the correct response side) and a spatial cue dimension (the side from which the sounds are played back), whereas the traditional joint Simon task uses two visual cue dimensions presented on a monitor (different colors that indicate the correct response side, and the depiction of a pointing hand, or an arrow, as interfering cue). Arguably, the different cue dimensions made the task easier; moreover, in the acoustic version, no *a* *priori* understanding of pointings or arrows had to be assumed, and also the broad spatial arrangement of task, stimuli and apparatus (real drawers as response options) versus stimulus presentation on a screen with simple buttons as response options may have contributed. Regardless of which factor made the task easier, the resulting demands were low enough that the joint Simon effect could be detected in the youngest age group that was able to participate in the task. Even in these two‐year‐olds, it could only be detected by the analysis of the more sensitive measure, namely subtle intention movements (first heading directions), but not in actual choices (manual responses). This suggests that already the two‐year‐olds, who demonstrably showed co‐representation effects in their intention movements, were able to suppress them in their actions.

How did human children reach this flexible adjustment when to merge and when not? Our data suggest they used mixed strategies: First, as the marmosets, they relied on mutual gaze as behavioral coordination smoother. Second, they also used verbal communication to mediate the uncertainty whose turn it was in the incompatible trials of the joint task condition. Finally, we also find an effect of inhibitory control, suggesting they also used more cognitive strategies to inhibit unwanted co‐representation. These results may contribute to explaining why humans excel in joint action and cooperation among primates: humans can rely not only on behavioral coordination smoothers to regulate joint action and cooperation, but can also rely on more cognitive strategies such as general inhibitory control and language. This falls in line with, and gives a different twist to, ideas that posit that language evolved to support human cooperation (e.g. Tomasello 2009).

The reliance on mutual gaze as coordination smoother not only in marmosets but also in human children may also inform views on the nature of co‐representation. Traditionally, the task/action co‐representation account (Sebanz et al. 2003) prevails, but the joint Simon effect can also be seen as the result of a response conflict as proposed in the agent co‐representation account (Wenke et al. 2011). Here, it is assumed that a stimulus indicating the right‐hand side response option does not prime the right‐hand side response, but rather primes the coactor sitting on the right‐hand side. This may arguably trigger a gaze toward this coactor and result in mutual gaze. Gaze directions may thus provide a useful tool in the future to further disentangle the nature of co‐representation in joint Simon tasks.

The present data from human children thus fall in line with the nonhuman primate data that better support the cognitive low‐level model of co‐representation (Figure 1). Co‐representation was present in all primate species and in rats (Katsu and Okanoya 2024), and it was not correlated with brain size (which among primates is a predictor for inhibitory control, MacLean et al. 2014, working memory, van Schaik et al. 2024, and general cognitive ability, Burkart et al. 2017; Deaner et al. 2007), and also not with intra‐individual variation in independently measured inhibitory control in Tonkean macaques, capuchin monkeys and marmoset monkeys (Miss, Sadoughi, et al. 2022). Co‐representation is thus most likely indeed a basic, cognitively undemanding process that does not require human‐like cognitive abilities as present in four‐year‐olds.

Co‐representation may not only be early emerging in both ontogeny and phylogeny, but also the default mode of encoding joint actions, perhaps as a consequence of how actions are encoded in the mammal brain (Bonini et al. 2022). Such an account is in line with research on infant inter‐subjectivity (Delafield‐Butt and Reddy 2025), and with altercentric accounts of the origin of social cognition, both in human ontogeny (Southgate 2020) and in phylogeny (Burkart and Southgate 2025). What all these accounts have in common is that they increasingly question the traditional premise in developmental psychology that human children begin life as egocentric beings (Kohlberg 1971; Piaget 1952), and only eventually manage to understand other social beings, as the result of an effortful, cognitively demanding process. Future work will reveal how this perspective will stand the test of time, both for the development and the evolution of social understanding.

This study has limitations. First, Swiss children are certainly WEIRD (Henrich et al. 2010) and not representative for other eco‐cultural contexts. Second, the sample sizes, in particular for the nonhuman primates, were small and did not include young immature monkeys, which are a particularly important test case for the ideas presented here. The sample size of children was equally small, in particular to reliably detect age effects, which will require further replication. Furthermore, the generality of the results to other species remains to be demonstrated empirically. Since the apparatus is simple and the test can easily be applied in a broad range of contexts, we hope that such data may become available in the near future.

In conclusion, co‐representation has originally been viewed as cognitively demanding, because effects were often only weak in adults and apparently emerged late during ontogeny. Our results however suggest it is upside down: co‐representation is most likely a very basic, cognitively undemanding mechanism, present in all primates tested so far and also in rats, and in human children as early as they are able to participate. The real crux turns out to be when to merge, or not. This is not primarily achieved with advanced cognitive abilities in primates and young children (Model 1 in Figure 1), but appears more fundamentally the result of social interaction dynamics where during routine joint action dyadic coordination smoothers are key (Model 2 in Figure 1), with more cognitive control becoming relevant only in older children. Intriguingly, these results are well in line with recent altercentric approaches to cognitive development that likewise suggest that human infants may start out by aligning with others (Southgate 2020), and increasing evidence suggests a similar pattern for cognitive evolution (Burkart and Southgate 2025).

## Author Contributions

**Fabia M. Miss**: writing – original draft, writing – review and editing, investigation, formal analysis; methodology, conceptualization. **Luxshiha Santharuban**: investigation, writing – review and editing. **Erik P. Willems**: writing – review and editing, formal analysis. **Judith M. Burkart**: writing – original draft, writing – review and editing, formal analysis, conceptualization, methodology.

## Funding

This work was supported by the Swiss National Science Foundation (grant no. 31003A_172979), the NCCR Evolving Language, Swiss National Science Foundation (agreement no. 51NF40_180888), and the European Research Council (ERC) under the European Union's Horizon 2020 research and innovation programme (grant agreement No 101001295).

## Ethics Statement

This research received approval from the Ethics Committee of the Faculty of Arts and Social Sciences of the University of Zurich. Written informed consent was obtained for all participants from a parent or legal guardian.

## Conflicts of Interest

All authors declare no conflicts of interest.

## Supporting information




**Supporting Materials**: desc70199‐sup‐0001‐SuppMat.docx

## Data Availability

All data needed to evaluate the conclusions in the paper are present in the paper and/or the Supplementary Information. A preprint of this study is available on OSF: https://doi.org/10.31234/osf.io/p7v4c

## References

[desc70199-bib-0001] Bartoń, K. 2025. MuMIn: Multi‐Model Inference. R Package Version 1.48.11. https://cran.r‐project.org/package=MuMIn.

[desc70199-bib-0002] Bates, D. , M. Mächler , B. M. Bolker , and S. C. Walker . 2015. “Fitting Linear Mixed‐Effects Models Using lme4.” Journal of Statistical Software 67, no. 1: 18637. 10.18637/jss.v067.i01.

[desc70199-bib-0003] Bonini, L. , C. Rotunno , E. Arcuri , and V. Gallese . 2022. “Mirror Neurons 30 Years Later: Implications and Applications.” Trends in Cognitive Sciences 26, no. 9: 767–781. 10.1016/j.tics.2022.06.003. 35803832

[desc70199-bib-0004] Burkart, J. M. , O. Allon , F. Amici , et al. 2014. “The Evolutionary Origin of Human Hyper‐Cooperation.” Nature Communications 5, no. 1: 4747. 10.1038/ncomms5747. 25158760

[desc70199-bib-0005] Burkart, J. M. , S. B. Hrdy , and C. P. van Schaik . 2009. “Cooperative Breeding and Human Cognitive Evolution.” Evolutionary Anthropology 18, no. 5: 175–186. 10.1002/evan.20222.

[desc70199-bib-0006] Burkart, J. M. , and K. Rueth . 2013. “Preschool Children Fail Primate Prosocial Game Because of Attentional Task Demands.” PLoS ONE 8, no. 7. 10.1371/journal.pone.0068440. PMC3700944 23844201

[desc70199-bib-0007] Burkart, J. M. , M. N. Schubiger , and C. P. van Schaik . 2017. “The Evolution of General Intelligence.” Behavioral and Brain Sciences 40: e195. 10.1017/S0140525x16000959. 27464851

[desc70199-bib-0008] Burkart, J. M. , and V. Southgate . 2025. “An Evolutionary Perspective on Altercentrism.” Neuroscience and Biobehavioral Reviews 176: 106280. 10.1016/j.neubiorev.2025.106280. 40639668

[desc70199-bib-0009] Ciani, F. , S. Dall'Olio , R. Stanyon , and E. Palagi . 2012. “Social Tolerance and Adult Play in Macaque Societies: A Comparison With Different Human Cultures.” Animal Behaviour 84, no. 6: 1313–1322. 10.1016/j.anbehav.2012.09.002.

[desc70199-bib-0010] Deaner, R. O. , K. Isler , J. M. Burkart , and C. van Schaik . 2007. “Overall Brain Size, and Not Encephalization Quotient, Best Predicts Cognitive Ability Across Non‐Human Primates.” Brain, Behavior and Evolution 70, no. 2: 115–124. 10.1159/000102973. 17510549

[desc70199-bib-0011] Delafield‐Butt, J. , and V. Reddy . 2025. Intersubjective Minds: Rhythm, Sympathy, and Human Being. Oxford University Press.

[desc70199-bib-0012] de Waal, F. B. M. 2000. “Attitudinal Reciprocity in Food Sharing Among Brown Capuchin Monkeys.” Animal Behaviour 60, no. 2: 253–261. 10.1006/anbe.2000.1471. 10973728

[desc70199-bib-0013] Dittrich, K. , A. Rothe , and K. C. Klauer . 2012. “Increased Spatial Salience in the Social Simon Task: A Response‐Coding Account of Spatial Compatibility Effects.” Attention, Perception, & Psychophysics 74: 911–929. 10.3758/s13414-012-0304-1. 22528612

[desc70199-bib-0014] Dolk, T. , B. Hommel , L. S. Colzato , S. Schütz‐Bosbach , W. Prinz , and R. Liepelt . 2014. “The Joint Simon Effect: A Review and Theoretical Integration.” Frontiers in Psychology 5: 974. 10.3389/fpsyg.2014.00974. 25249991 PMC4155780

[desc70199-bib-0015] Dudarev, V. , G. Iarocci , and J. T. Enns . 2021. “A Joint Simon Effect in Children Diagnosed With ASD is Expressed Differently From Neurotypical Children and Adults.” Visual Cognition 30, no. 1–2: 29–41. 10.1080/13506285.2021.1958039.

[desc70199-bib-0016] Fox, J. , and S. Weisberg . 2019. An R Companion to Applied Regression. 3rd ed. Sage. https://www.john‐fox.ca/Companion/.

[desc70199-bib-0017] Gerstadt, C. L. , Y. J. Hong , and A. Diamond . 1994. “The Relationship Between Cognition and Action: Performance of Children 3 1/2 –7 Years Old on a Stroop‐ Like Day‐Night Test.” Cognition 53, no. 2: 129–153. 10.1016/0010-0277(94)90068-X. 7805351

[desc70199-bib-0018] Henrich, J. , S. J. Heine , and A. Norenzayan . 2010. “Beyond WEIRD: Towards a Broad‐Based Behavioral Science.” Behavioral and Brain Sciences 33, no. 2–3: 111–135. 10.1017/S0140525x10000725. 20550733

[desc70199-bib-0019] Iani, C. , F. Anelli , R. Nicoletti , L. Arcuri , and S. Rubichi . 2011. “The Role of group membership on the modulation of joint action.” Experimental Brain Research 211, no. 3–4: 439–445. 10.1007/s00221-011-2651-x. 21472442

[desc70199-bib-0020] Iani, C. , and S. Rubichi . 2025. “Acting and Practising Together: Modulations of the Joint Simon Effect in 6‐ to 8‐year‐Old Children.” Infant and Child Development 34, no. 4. 10.1002/icd.70044.

[desc70199-bib-0021] Katsu, N. , and K. Okanoya . 2024. “Examination of the Joint Simon Effect in Rats: Changes in Task Performance Based on Actions of the Partner.” Behavioural Processes 216: 105005. 10.1016/j.beproc.2024.105005. 38365010

[desc70199-bib-0022] Kiernan, D. , M. Ray , and T. N. Welsh . 2012. “Inverting the Joint Simon Effect by Intention.” Psychonomic Bulletin & Review 19: 914–920. 10.3758/s13423-012-0283-1. 22718258

[desc70199-bib-0023] Kohlberg, L. 1971. “Stages of Moral Development as a Basis for Moral Education.” In Moral *E*ducation: Int*erdisciplinary Approaches* . University of Toronto Press.

[desc70199-bib-0024] Korkman, M. , U. Kirk , and S. Kemp . 1998. Nepsy: A Developmental Neuropsychological Assessment. Psychological Corporation.

[desc70199-bib-0025] Kristen, S. , C. Thoermer , T. Hofer , G. Aschersleben , and B. Sodian . 2006. “Skalierung Von “Theory of Mind”‐Aufgaben.” Zeitschrift Für Entwicklungspsychologie Und Pädagogische Psychologie 38, no. 4: 186–195. 10.1026/0049-8637.38.4.186.

[desc70199-bib-0026] Krupenye, C. , and J. Call . 2019. “Theory of Mind in Animals: Current and Future Directions.” Wiley Interdisciplinary Reviews: Cognitive Science 10, no. 6: 1–25. 10.1002/wcs.1503. 31099977

[desc70199-bib-0027] Lin‐Lin, L. I. N. , L. I. U. Wen , and G. O. N. G. Beng . 2021. “The Development of Preschool Children's Co‐Representation in Joint Actions.” Journal of Psychological Science 1: 60.

[desc70199-bib-0028] MacLean, E. L. , B. Hare , C. L. Nunn , et al. 2014. “The Evolution of Self‐Control.” Proceedings of the National Academy of Sciences 111, no. 20: E2140–E2148. 10.1073/pnas.1323533111. PMC4034204 24753565

[desc70199-bib-0029] Martínez, M. , M. H. Babb , F. Range , and S. F. Brosnan . 2024. “The Joint Simon Task is not Joint for Capuchin Monkeys.” Scientific Reports 14, no. 1. 10.1038/s41598-024-55885-x. PMC10928181 38467698

[desc70199-bib-0030] McClung, J. S. , S. Placì , A. Bangerter , F. Clément , and R. Bshary . 2017. “The Language of Cooperation: Shared Intentionality Drives Variation in Helping as a Function of Group Membership.” Proceedings of the Royal Society B: Biological Sciences 284, no. 1863: 20171682. 10.1098/rspb.2017.1682. PMC5627217 28931743

[desc70199-bib-0031] Mendres, K. A. , and F. B. M. de Waal . 2000. “Capuchins do Cooperate: The Advantage of an Intuitive Task.” Animal Behaviour 60, no. 4: 523–529. 10.1006/anbe.2000.1512. 11032655

[desc70199-bib-0032] Milward, S. J. , S. Kita , and I. A. Apperly . 2014. “The Development of Co‐Representation Effects in a Joint Task: Do Children Represent a Co‐Actor?” Cognition 132, no. 3: 269–279. 10.1016/j.cognition.2014.04.008. 24853630

[desc70199-bib-0033] Milward, S. J. , S. Kita , and I. A. Apperly . 2017. “Individual Differences in Children's Co‐representation of Self and Other in Joint Action.” Child Development 88, no. 3: 964–978. 10.1111/cdev.12693. 27966800

[desc70199-bib-0034] Miss, F. M. , J. E. C. Adriaense , and J. M. Burkart . 2022. “Towards Integrating Joint Action Research: Developmental and Evolutionary Perspectives on Co‐Representation.” Neuroscience and Biobehavioral Reviews 143: 104924. 10.1016/j.neubiorev.2022.104924. 36283538

[desc70199-bib-0035] Miss, F. M. , and J. M. Burkart . 2018. “Co‐representation During Joint Action in Marmoset Monkeys (*Callithrix jacchus*).” Psychological Science 29, no. 6: 984–995. 10.1177/0956797618772046. 29702031

[desc70199-bib-0036] Miss, F. M. , H. Meunier , and J. M. Burkart . 2022. “Primate Origins of Co‐representation and Cooperative Flexibility: A Comparative Study With Common Marmoset (*Callithrix jacchus*), Brown Capuchins (*Sapajus apella*), and Tonkean Macaques (*Macaca tonkeana*).” *Journal of Comparative Psychology* . 10.1037/com0000315. 35389713

[desc70199-bib-0037] Miss, F. M. , B. Sadoughi , H. Meunier , and J. M. Burkart . 2022. “Individual Differences in Co‐Representation in Three Monkey Species (*Callithrix jacchus*, *Sapajus apella*, and *Macaca tonkeana*) in the Joint Simon Task: The Role of Social Factors and Inhibitory Control.” Animal Cognition 25: 1399–1415. 10.1007/s10071-022-01622-8. 35508572 PMC9652238

[desc70199-bib-0038] Perry, S. , and L. Rose . 1994. “Begging and Transfer of Coati Meat by White‐Faced Capuchin Monkeys, *Cebus Capucinus* .” Primates 35, no. 4: 409–415. 10.1007/BF02381950.

[desc70199-bib-0039] Petit, O. , C. Desportes , and B. Thierry . 1992. “Differential Probability of “Coproduction” in Two Species of Macaque (*Macaca* tonkeana, M. mulatta).” Ethology 90, no. 2: 107–120. 10.1111/j.1439-0310.1992.tb00825.x.

[desc70199-bib-0040] Petit, O. , and B. Thierry . 1994. “Aggressive and Peaceful Interventions in Conflicts in Tonkean Macaques.” Animal Behaviour 48, no. 6: 1427–1436. 10.1006/anbe.1994.1378.

[desc70199-bib-0041] Pfister, R. , T. Dolk , W. Prinz , and W. Kunde . 2014. “Joint Response–Effect Compatibility.” Psychonomic Bulletin & Review 21: 817–822. 10.3758/s13423-013-0528-7. 24101572

[desc70199-bib-0042] Piaget, J. 1952. The Origin of Intelligence in Children. International Universities Press.

[desc70199-bib-0043] R Core Team . 2019. R: A Language and Environment for Statistical Computing. R Foundation for Statistical Computing. http://www.r‐project.org/.

[desc70199-bib-0044] Rakoczy, H. 2022. “Foundations of Theory of Mind and Its Development in Early Childhood.” Nature Reviews Psychology 1, no. 4: 223–235. 10.1038/s44159-022-00037-z.

[desc70199-bib-0045] Ruys, K. I. , and H. Aarts . 2010. “When Competition Merges People's Behavior: Interdependency activates shared action representations.” *Journal of* Experimental Social Psychology 46, no. 6: 1130–1133. 10.1016/j.jesp.2010.05.016.

[desc70199-bib-0046] Saby, J. N. , C. A. Bouquet , and P. J. Marshall . 2014. “Young Children Co‐Represent a Partner's Task: Evidence for a Joint Simon Effect in Five‐Year‐Olds.” Cognitive Development 32: 38–45. 10.1016/j.cogdev.2014.08.001.

[desc70199-bib-0047] Sahaï, A. , A. Desantis , O. Grynszpan , E. Pacherie , and B. Berberian . 2019. “Action Co‐Representation and the Sense of Agency During a Joint Simon Task: Comparing Human and Machine Co‐Agents.” Consciousness and Cognition 67: 44–55. 10.1016/j.concog.2018.11.008. 30522081

[desc70199-bib-0048] Schaffner, C. M. , and N. G. Caine . 2000. “The Peacefulness of Cooperatively Breeding Primates.” In Natural Conflict Resolution, edited by F. Aureli and F. B. M. de Waal , 155–169. University of California Press.

[desc70199-bib-0049] Sebanz, N. , G. Knoblich , and W. Prinz . 2003. “Representing Others' Actions: Just Like One's Own?” Cognition 88, no. 3: B11–B21. 10.1016/S0010-0277(03)00043-X. 12804818

[desc70199-bib-0050] Shafaei, R. , Z. Bahmani , B. Bahrami , and M. Vaziri‐Pashkam . 2020. “Effect of Perceived Interpersonal Closeness on the Joint Simon Effect in Adolescents and Adults.” Scientific Reports 10, no. 1: 1–10. 10.1038/s41598-020-74859-3. 33093544 PMC7582195

[desc70199-bib-0051] Snowdon, C. T. , and K. A. Cronin . 2007. “Cooperative Breeders do Cooperate.” Behavioural Processes 76, no. 2: 138–141. 10.1016/j.beproc.2007.01.016. 17703900 PMC2080785

[desc70199-bib-0052] Song, X. , M. Dong , K. Feng , J. Li , X. Hu , and T. Liu . 2024. “Influence of Interpersonal Distance on Collaborative Performance in the Joint Simon Task—An fNIRS‐Based Hyperscanning Study.” Neuroimage 285, no. 99: 120473. 10.1016/j.neuroimage.2023.120473. 38040400

[desc70199-bib-0053] Southgate, V. 2020. “Are Infants Altercentric? The Other and the Self in Early Social Cognition.” Psychological Review 127, no. 4: 505–523. 10.1037/rev0000182. 31868391

[desc70199-bib-0054] Thierry, B. 2007. “Unity in Diversity: Lessons From Macaque Societies.” Evolutionary Anthropology 16, no. 6: 224–238. 10.1002/evan.20147.

[desc70199-bib-0055] Thierry, B. , J. R. Anderson , C. Demaria , C. Desportes , and O. Petit . 1994. “Tonkean Macaque Behaviour From the Perspective of the Evolution of Sulawesi Macaques.” In Current Primatology, Social Development, Learning and Behaviour, edited by J. J. Roeder , B. Thierry , J. R. Anderson , and N. Herrenschmidt , 2: 103–117. Université Louis Pasteur.

[desc70199-bib-0056] Tomasello, M. 2009. Why We Cooperate. MIT Press.

[desc70199-bib-0057] Tsai, C.‐C. , W.‐J. Kuo , D. L. Hung , and O. J. L. Tzeng . 2008. “Action Co‐Representation is Tuned to Other Humans.” Journal of Cognitive Neuroscience 20, no. 11: 2015–2024. 10.1162/jocn.2008.20144. 18416679

[desc70199-bib-0058] van Schaik, C. P. , I. Jacobs , J. M. Burkart , et al. 2024. “Short‐Term Memory, Attentional Control and Brain Size in Primates.” Royal Society Open Science 11, no. 5: 231541. 10.1098/rsos.231541. 39076802 PMC11285803

[desc70199-bib-0059] Weaver, A. , and F. B. M. de Waal . 2003. “The Mother‐Offspring Relationship as a Template in Social Development: Reconciliation in Captive Brown Capuchins (*Cebus apella*).” Journal of Comparative Psychology 117, no. 1: 101–110. 10.1037/0735-7036.117.1.101. 12735370

[desc70199-bib-0060] Wellman, H. M. , and D. Liu . 2004. “Scaling of Theory‐of‐Mind Tasks.” Child Development 75, no. 2: 523–541. 10.1111/j.1467-8624.2004.00691.x. 15056204

[desc70199-bib-0061] Wenke, D. , S. Atmaca , A. Holländer , R. Liepelt , P. Baess , and W. Prinz . 2011. “What is Shared in Joint Action? Issues of Co‐Representation, Response Conflict, and Agent Identification.” Review of Philosophy and Psychology 2, no. 2: 147–172. 10.1007/s13164-011-0057-0.

[desc70199-bib-0062] Wickham, H. 2016. ggplot2: Elegant Graphics for Data Analysis. Springer Verlag. https://ggplot2.tidyverse.org.

[desc70199-bib-0063] Yamaguchi, M. , T. N. Welsh , K. C. Klauer , and K. Dittrich . 2019. “Editorial: What's Shared in Sharing Tasks and Actions? Processes and Representations Underlying Joint Performance.” Frontiers in Psychology 10: 659. 10.3389/fpsyg.2019.00659. 31001162 PMC6453998

