# Supplementary material for: To Merge or not: The Early Onto‐ and Phylogenetic Origin of Co‐Representation
Source: Dev Sci. 2026 Apr 26;29:e70199. doi: 10.1111/desc.70199 (PMC13111789; doi:10.1111/desc.70199)
Supplement: Supplementary file 1 — Supporting Materials: desc70199‐sup‐0001‐SuppMat.docx [file DESC-29-e70199-s001.docx]

Supplementary Information for

**To merge or not: The onto- and phylogenetic origin of co-representation**

**This file includes:**

1. Supplementary Methods
   1. Procedure of the Simon task
   2. Details of the ToM and inhibition tasks
   3. Behavioral definitions for data coding
2. Supplementary Data
   1. The individual and the joint Simon effect in response choices
   2. Correlation with ToM and IC
   3. Communicative cues in children
3. Supplementary Figure S1 – S9
4. Supplementary Table S1 – S3
5. **Supplementary Methods**
   1. **Procedure of the Simon task**

The children were first familiarized with the experimenter(s) (E, either one or two) and the experimental set-up including the functioning of the testing device, the playpen, the curtain, and the partition grid. In one to two demonstration sessions, E explained and demonstrated the task procedure to small groups of participants. Subsequently, the children could do a few trials themselves individually or in pairs while the rest of the group was observing. In the following training phase, the participants individually learned the association between the stimulus (sound “L” or “R”) and the corresponding response side on the device (the left-hand or the right-hand drawer). In this phase, the sounds were broadcast from a central position and thus did not create any stimulus incompatibility. Before the start of a trial, the participant was asked to sit in the middle of the playpen indicated with the cross on the floor and to look at the smiling face attached to the curtain in front.

A trial began with the emitted sound while the curtain was opened simultaneously, and it ended when the participant (or one of the two participants in the dyadic condition, see below) pulled one of the two drawers, thus asking for an exclusive choice in each trial. In case of a correct choice, the participant could retrieve the reward, while the other cup was opened to show the non-baited side. In case of an incorrect choice, the other cup was opened to show the baited side and then the curtain was closed. Training sessions lasted for 1 – 17 trials depending on the participants’ motivation. As soon as the participants reached the testing criterion of either 100 % correct choices in a training session of 8 trials, or at least 80 % correct choices in a training session of 10 – 12 trials, they were introduced to the joint task condition (training version in which the sounds were still emitted from a central position). As soon as the dyads reached the testing criterion of either 100 % correct choices in a training session of 6 trials, or at least 80 % correct choices in a training session of 10 – 12 trials, they moved on to the testing phase containing all task conditions (full, half, joint and joint-control task).

Every test session started with two to four reminder pre-trials with the sounds emitted from a central position. The procedure was identical to the training. The criterion for starting a test session on the particular day was to choose each baited side correctly at least once. In the test trials, the sounds were broadcast from either the left- or the right- hand side, creating stimulus incompatibility in half of the trials. The procedure remained identical, except for possible trial endings in the half and joint-control task conditions, in which only one response side was accessible. A persistent reach towards the cup in the middle, a pointing gesture (i.e. an extended arm [either fully or slightly bent] with the index finger or open hand directed to the inaccessible side, Liszkowski et al., 2006) or a verbal reference to the inaccessible side were considered as a trial ending. In such a case, E asked the participant for a confirmation of her choice, opened the other cup after approximately 10 s (corresponding to three times as long as the participants’ mean latencies to grab the handle in the half and the joint-control task) and transferred the reward to the child in case of a correct choice.

- 1. **Details of the ToM and inhibition tasks**

According to the children’s language preferences, the tasks were explained and conducted in (Swiss-) German or in English. Depending on the child’s motivation, the ToM and inhibition tasks were administered in one single session or split into two sessions on two days. Every child individually participated once in each of these tasks.

***ToM tasks***

The five ToM tests (diverse desire test, diverse belief test, knowledge access test, explicit false belief test, contents false belief test, see below for details and Fig. S2) contained short stories that were narrated with the aid of pictures showing the items and situations, and Playmobil figures representing the protagonists. Every test consisted of a target question about the behavior or mental state of the protagonist, and tests No. 3, 4 and 5 additionally contained a control question in form of a memory or a reality question.

1. Diverse desire test

E presented a Playmobil figure, “Herr Müller”, who had the choice between two food items for snack time, a carrot and a cookie, both illustrated on a sheet of paper. First, E asked the participant about his/her preferred snack and then told the participant that Herr Müller preferred the other one. Subsequently, E asked the target question which snack Herr Müller would choose. A point was scored if the participant answered the target question contrary to his/her answer to the own-desire question.

1. Diverse belief test

E presented a Playmobil figure, Julia, who wanted to find her cat hidden either in the bushes or in the garage, both illustrated on a sheet of paper. First, E asked the participant where she/he thought the cat was hiding and then told the participant that Julia believed it to be in the other location. Subsequently, E asked the target question where Julia would search for her cat. A point was scored if the participant answered the target question contrary to his/her answer to the own-belief question.

1. Knowledge access test

E presented a box and asked the participant what she/he thought was inside [any answer was possible]. E opened the box, revealing a little stuffed panda, and then closed the box again with the stuffed panda inside. E asked a memory question to ensure the participant knew about the box’s content. Next, E presented an approaching playmobil figure, Anna, who had never looked inside the box. Subsequently, E asked the target question if Anna knew what was inside the box, followed by a control question if Anna had looked inside the box. A point was scored if the participant answered both, the target question and the control question with “no”.

1. Explicit false belief test

E presented a Playmobil figure, Paul, who was searching for his gloves that could be either in the backpack or in the wardrobe, both illustrated on a sheet of paper. E told the participant that the gloves were in the backpack but that Paul thought they were in the wardrobe. Subsequently, E asked the target question where Paul would search his gloves, followed by a control question where his gloves really were. A point was scored if the participant answered the target question with “wardrobe” and the control question with “backpack”.

1. Contents false belief test

E presented a smarties package and asked the participant what she/he thought was inside the package. In case the participant did not answer with “smarties”, some additional questions were asked [What kind of package is this? Do you think there are some books inside?...] until the participant answered with “smarties”. Then, E opened the package, revealing a little plastic dog, and closed the package again with the plastic dog inside. E asked a memory question to ensure the participant knew about the package’s content. Next, E presented an approaching playmobil figure, Lukas, who had never looked inside the package. Subsequently, E asked the target question what Lukas thought was inside the package, followed by a control question if Lukas had looked inside the package. A point was scored if the participant answered the target question with “smarties” and the control question with “no”.

***Inhibition tasks***

To ensure task understanding in the day-night Stroop task, the children were first asked with a control question to choose one of the images (Fig. S3a) according to the word pronounced. If the control question was answered correctly, they continued with 12 test trials. In case of an incorrect answer, the task was explained again, and the child completed up to six practice trials with repeated task explanations in between if needed before the completion of 12 test trials. In the test trials, the two words were selected pseudo-randomly but never more than twice in a row with a total of six trials for each word and no time limit on responses.

In the statue task, the position was to stand upright on both feet with one arm parallel to the body and the hand touching a surface to hold on to and the other arm bended at the elbow resulting in the forearm being perpendicular to the body (Fig. S3b). In predefined time intervals, E made a series of distracting noises (dropping a pencil, clapping or coughing once, knocking twice, saying “hum, hum”). In 5 s intervals, observations were made for errors (i.e. body movements, opening the eyes, talking or laughing). If the child showed one of these behaviors during the task, E shortly reminded her to stay still and/ or to keep her eyes closed.

- 1. **Behavioral definitions for data coding**

***The Simon task***

To code the first heading direction, we observed if the focal participant first showed a body orientation or subtle movement to the left-hand or the right-hand side, which was defined as one of the following behaviors:

1. planting a hand or a foot and leaning the upper body to the left or right;
2. extending an arm or a leg (partially or completely) and leaning the upper body to the left or right;
3. covering some distance to the left or right on one’s knees or feet or by dragging the buttocks;
4. a pointing gesture or verbal reference directed to the inaccessible response side in the half and the joint-control task.

In the social task conditions (i.e. joint and joint-control task), we also coded (as yes or no per trial and category) whether during the trial (i.e. cases in which the behavior had started already before the onset of the stimulus were not considered) the two participants

1. gazed at each other simultaneously (i.e. mutual gaze), and
2. showed verbal and/or gestural expressions belonging to one of the following categories:
   - *ask*, i.e. a question declared as a pointing gesture or a verbal expression in order to receive a hint about the correct response side (e.g. point and “Is it here?”, point and “Go that way?”, “This side?”, “Oh, is there something underneath?”, point and “Is it this one, no?”, “Do you know which one?”, “Is it my turn?”)
   - *exclaim*, i.e. declare uncertainty by expressing surprise, hesitation, confusion, or a doubt (e.g. “I don’t know”, “Häähh”, “Wow!”, “Uuuuhhh”, “I’m confused”, “Huch!”)
   - *inform*, i.e. declare certainty by
     1. expressing a confirmation, an insight or knowledge – including a call upon the partner to act – (e.g. “It’s your turn!”, “It’s yours, [partner’s name]!”, “Yes” and nodding, “Aha!”, “I get this one!”, “I know where it is!”, “It’s for me”), or
     2. making a gestural and/or verbal reference to a response side (e.g. “On this side, I know it!”, “Again this side!”, point and “It was here”, point and “This music!”, “It’s over there!”, point and “There!”, “On your side, [partner’s name]!”, point and “It’s for you!”, “It was over there, see!” and point)
   - *correct*, i.e. a self- or partner-directed verbal expression in the negative to correct the current state of decision making, possibly combined with a gestural reference to the response side (e.g. “I can’t go that way!”, “Not here!”, “No”, point and shake the head, “No, on the other side”, “Not this”, “No, it’s my turn!”, “No, pull there!” and point, “No, it was on my side!”)

***The statue task***

For the statue task (Korkman et al., 1998, NEPSY statue task), errors were defined as

1. body movements (moving the arm up or down more than 45°, turning the head or the body, lifting one leg, swinging it, or scraping the floor, walking, bending the knees, passing the hand over the face or the hair),
2. opening the eyes,
3. talking or laughing.

A silent smile or subtle movement of the fingers or the toes were not counted as errors. A score of 2 was coded for each 5 s interval in which there occurred no errors; a score of 1 was coded for each 5 s interval in which there occurred one error; a score of 0 was coded for each 5 s interval in which there occurred two or more errors. If the child interrupted the task before the end and did not want to restart, a score of 0 was coded for all the remaining 5 s intervals.

1. **Supplementary Data**
   1. **The individual and the joint Simon effect in response choices**

The analysis of the participants’ response choices did not reveal a reliable individual and joint Simon effect which had predicted more incorrect response choices in incompatible than compatible trials in the full and the joint task only, but not in the half and the joint-control task (Fig. S5). Accordingly, the full model revealed a significant effect of compatibility, task, age and session [χ^2^_11_ = 75.73, p < 0.001, pseudo-R^2^_c_ = 0.47, ∆AIC = 53.7, N_total_ = 4979, N_individuals_ = 30], however adding the interaction term between task and compatibility did not improve the model fit (χ^2^_3_ = 1.14, p = 0.77, ∆AIC = 4.7). Incorrect choices occurred slightly more often in incompatible than compatible trials across the four task conditions, β ± SE = -0.36 ± 0.09, 95% confidence interval [CI] [-0.55, -0.18], z = -3.94, p < 0.001. Incorrect choices were marginally increased in the control tasks compared to the experimental tasks, β ± SE = 0.16 ± 0.06, 95% CI [0.05, 0.26], z = 2.81, p = 0.005. Younger participants made more incorrect response choices than older participants in all task conditions, β ± SE = 0.13 ± 0.02, 95% CI [0.08, 0.17], z = 5.79, p < 0.001. This age effect was particularly strong in incorrect choices in incompatible trials (Fig. S6). The linear trend of session (β ± SE = 0.64 ± 0.10, 95% CI [0.44, 0.84], z = 6.35, p < 0.001) revealed a learning effect over time. Incorrect response choices occurred slightly more often in the first session than in the last session in all task conditions (*full task* mean percentage 19.03 ± SE 3.67 *vs.* 8.80 ± SE 2.94; *half task* 16.97 ± SE 3.38 *vs.* 12.60 ± SE 3.14; *joint task* 12.54 ± SE 3.80 *vs.* 5.46 ± SE 2.33; *joint-control task* 19.58 ± SE 3.55 *vs.* 10.28 ± SE 3.14).

- 1. **Correlation with ToM and IC**

To test if the participants’ performance scores in the day-night Stroop task and in the statue task could be combined into a composite measure for inhibitory control ability, we converted the values into z scores and tested their relationship with a Spearman’s rank correlation analysis. Since the participants’ performance scores in both tasks showed a moderate to strong positive correlation (Spearman’s rho (r_s_) = 0.59, p < 0.001), we added the two scores together resulting in a composite inhibitory control score per individual ranging from 0 to 42. The higher the composite score, the stronger the inhibition ability.

Both, the ToM scores and the two inhibitory control scores (day-night Stroop and statue task scores) showed an increase with age (*ToM tasks*: Spearman’s rho (r_s_) = 0.57, p < 0.001, Fig. S7a; *Day-night Stroop task*: r_s_ = 0.42, p = 0.020, Fig. S7b; *Statue task*: r_s_ = 0.70, p < 0.001, Fig. S7c.

- 1. **Communicative cues in children**

When analyzing the trials of both social test conditions (joint and joint-control task) together, communicative cues were correlated with mutual gaze within the same trial (β ± SE = 1.99 ± 0.20, 95% CI [1.60, 2.38], z = 10.08, p < 0.001). Adding mutual gaze as a predictor variable thus significantly improved the model fit [χ^2^_1_ = 105.18, p < 0.001, pseudo-R^2^_c_ = 0.44, ∆AIC = 103.1, N_total_ = 2131, N_individuals_ = 30].

**c.**

**Outer cup**

**Middle cup**


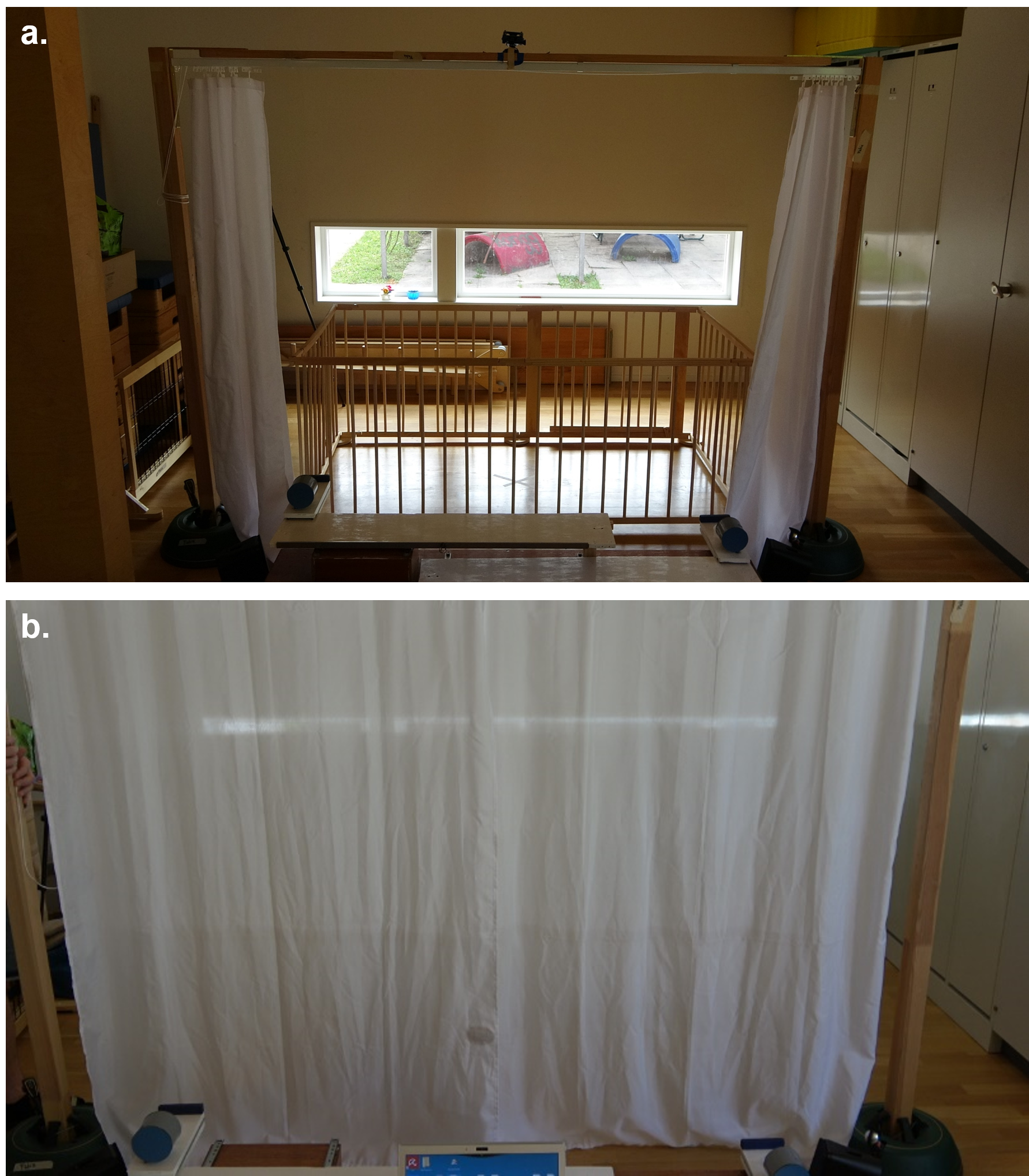


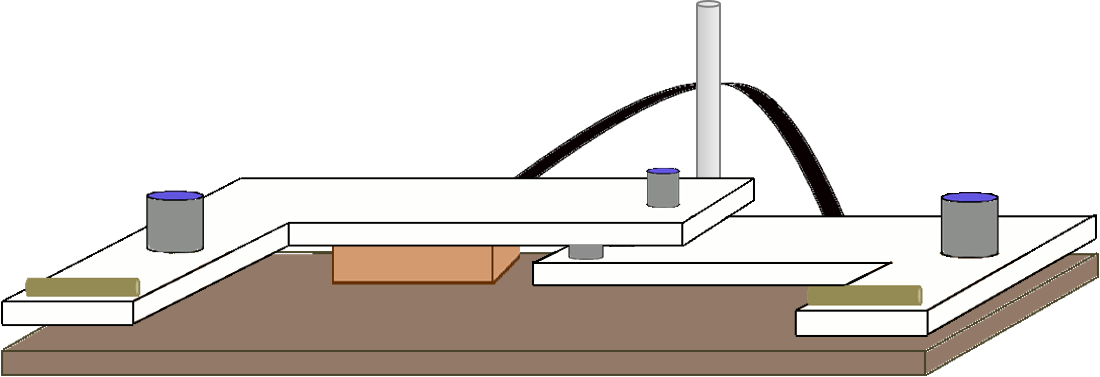


**Figure S1 Experimental set-up of the Simon task (full task condition)** showing **(a)** the cross on the floor in the middle of the playpen, the curtain attached to the wooden frame, and the testing device in the front, **(b)** the sticker showing a smiling face attached to the curtain in front of the child, and **(c)** the testing device with the two sliding drawers, each with a handle to grab, and the cups covering a potential reward. In the joint task, the active puller could flip over and retrieve the reward from the outer cup and the partner child from the middle cup. The cord connecting the back sides of the drawers ensured a backwards and out of reach movement of the nonchosen drawer as soon as one drawer was pulled.


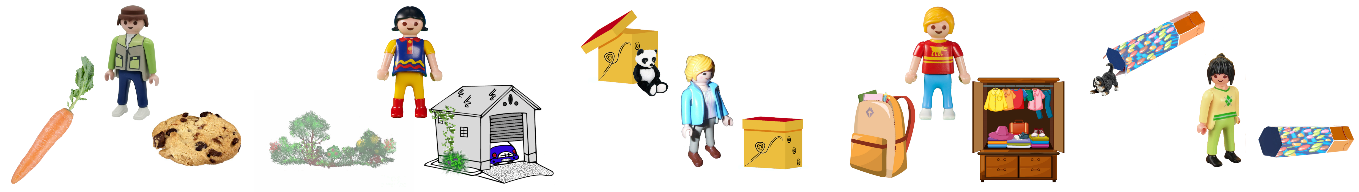


Image: brgfx on Freepik

Image: pch.vector on

Freepik


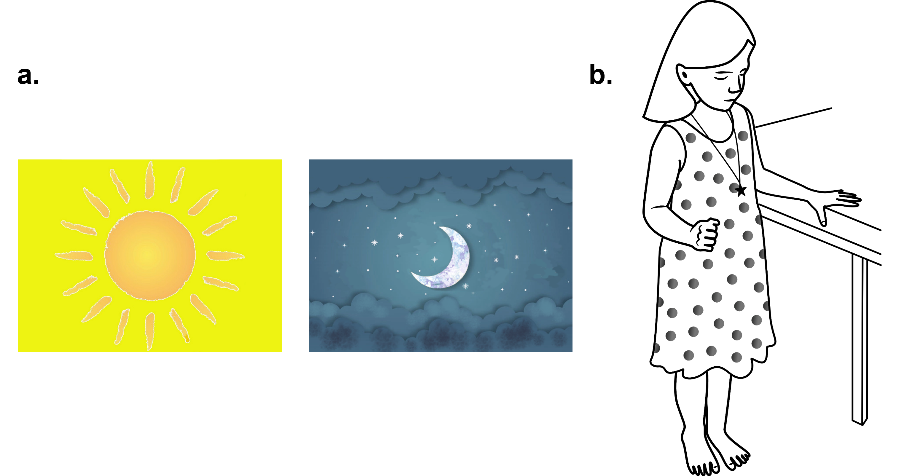


**Figure S2 ToM tests.** The tests were presented by their ascending level of difficulty (a) diverse desire test, (b) diverse belief test, (c) knowledge access test, (d) explicit false belief test, (e) contents false belief test.

**Figure S3 Inhibitory control tasks**. To measure motor inhibition and persistence, we used an adapted version of (a) the day-night Stroop task and (b) the statue task.

*

*

**Figure S4 Simon effect in first heading directions in the four task conditions.** The Simon effect is shown as the difference in the percentage of first movements towards the incorrect response side between incompatible and compatible trials, calculated per session and individual. Each point (in red) indicates a mean value per age. The boxes and whiskers represent medians and lower and upper quartile scores. The regression lines show the correlations between incorrect first heading directions and age. The shaded areas display 95% confidence intervals.


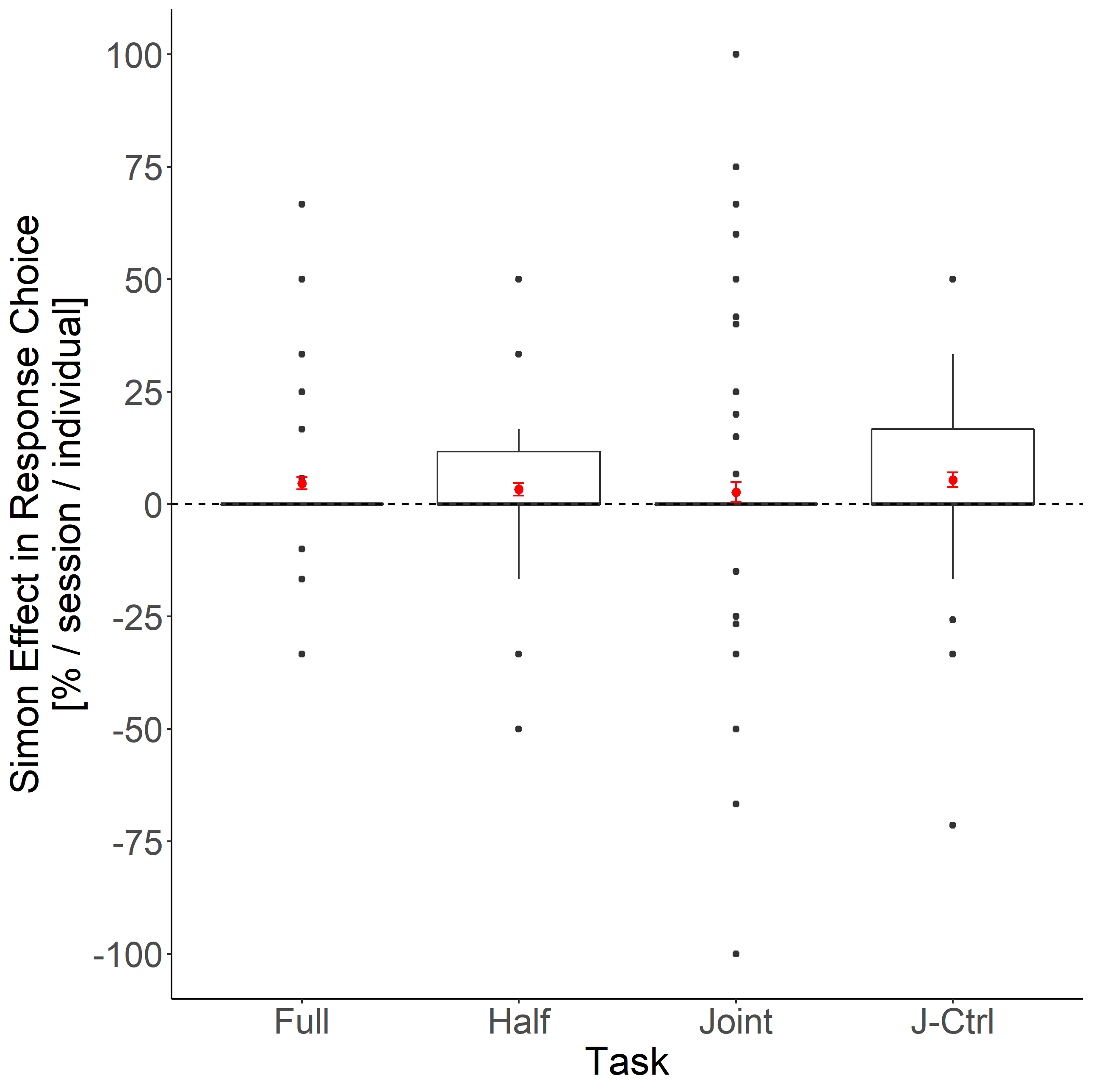


**Figure S5 Children’s Simon effect in response choices in the four task conditions.** The Simon effect is shown as the difference in the percentage of incorrect response choices between incompatible and compatible trials calculated per session and individual. The boxes and whiskers represent medians and lower and upper quartile scores. Error bars (in red) represent standard errors of the mean.


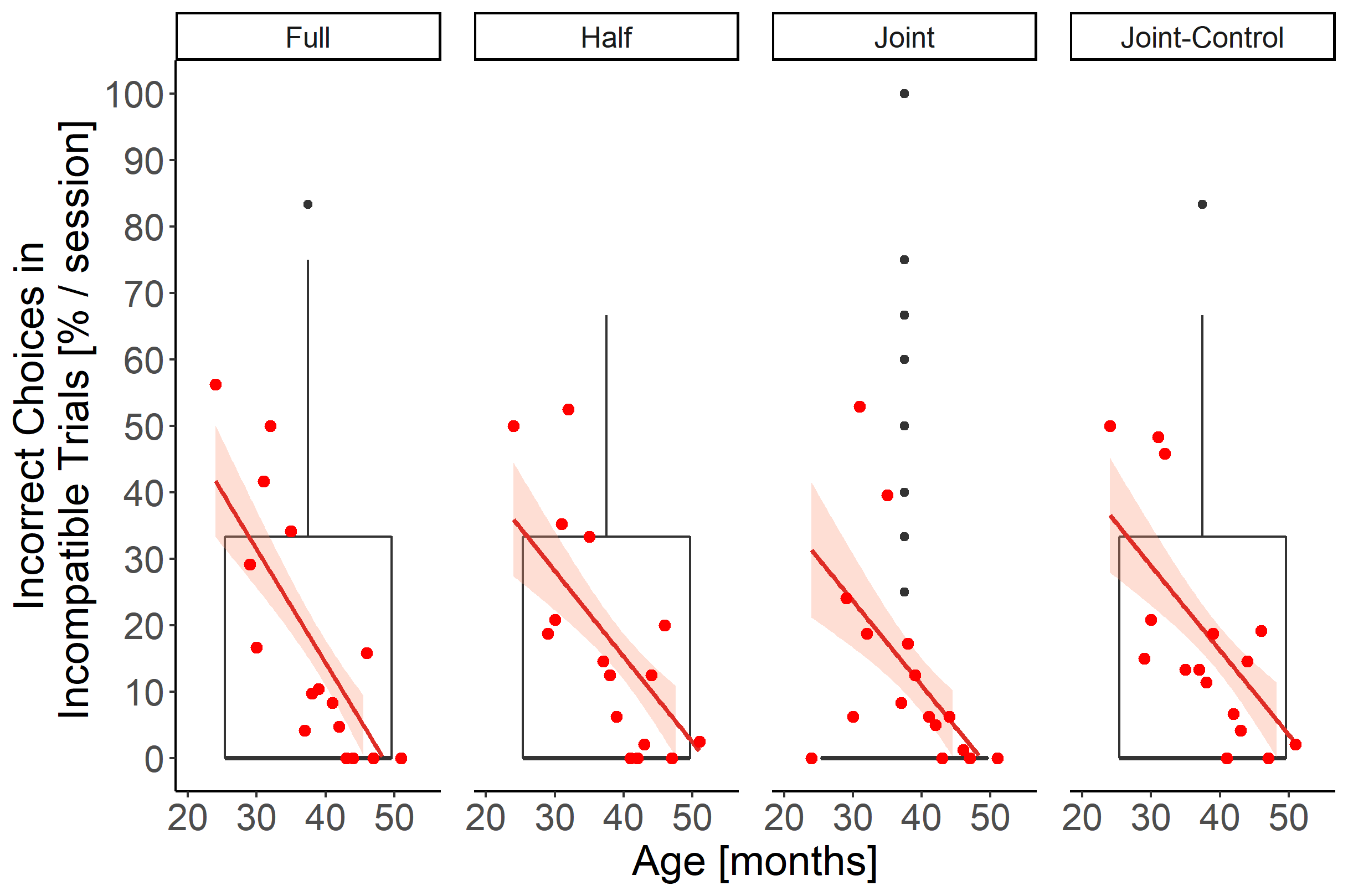


**Figure S6 Children’s incorrect choices in incompatible trials according to their age.** Mean percentages calculated per session of incorrect manual response choices in incompatible trials across the four task conditions. Each point (in red) indicates a mean value per age. The boxes and whiskers represent medians and lower and upper quartile scores.


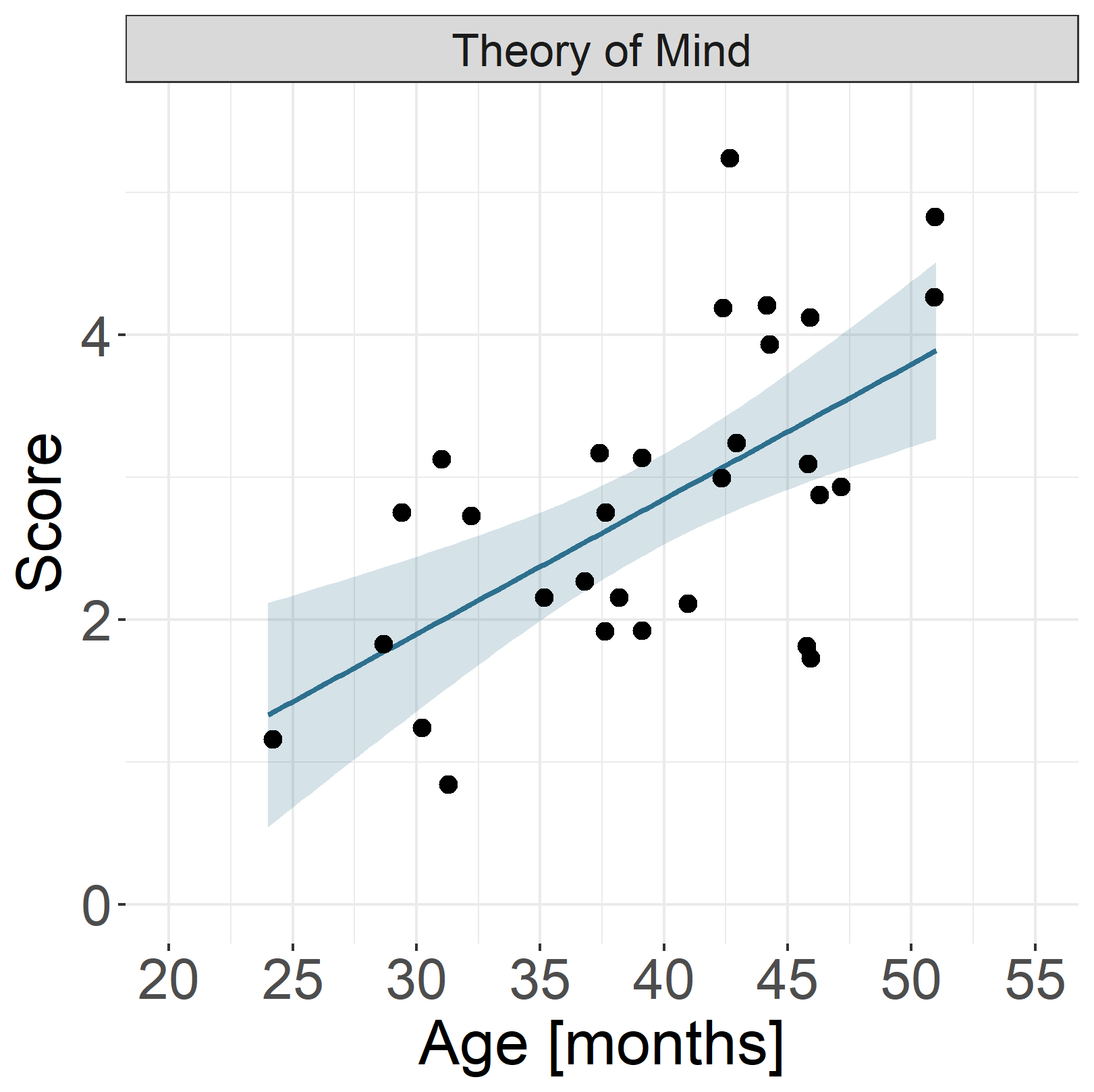

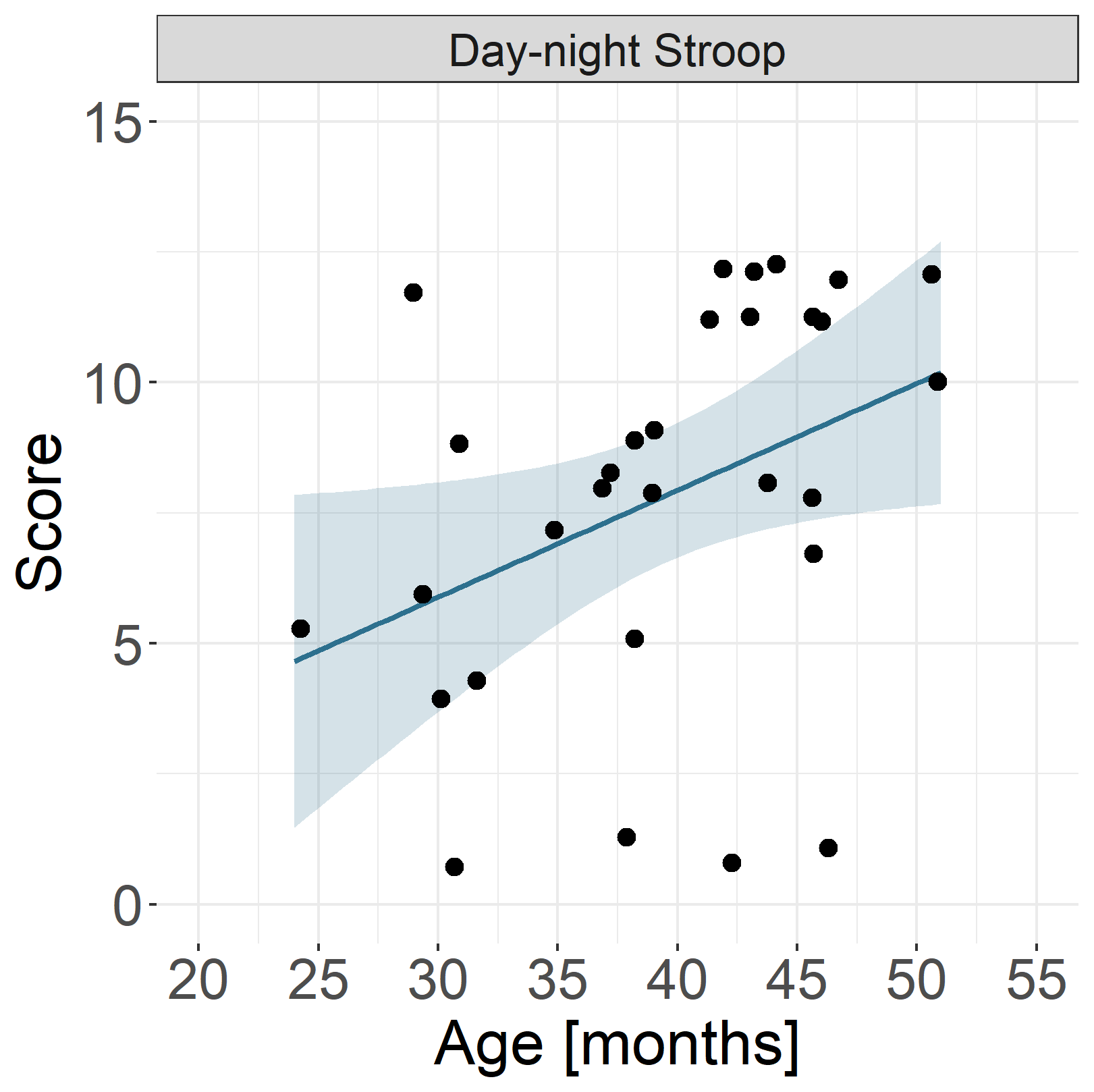

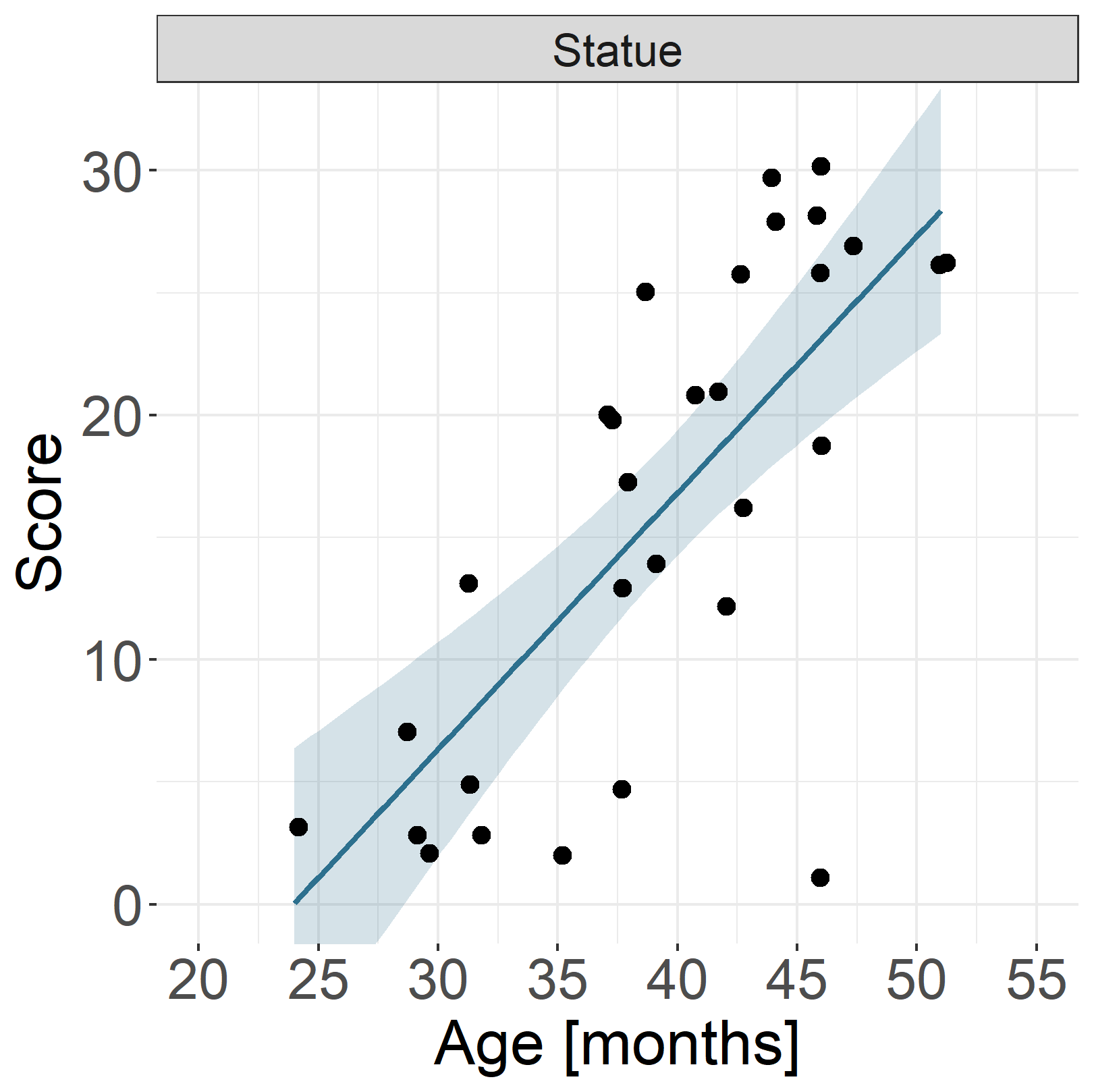


**Figure S7 ToM and inhibitory control abilities.** Relationship between the participants’ performance in (a) the ToM task with a score ranging from 0 to 5, and age (b) the day-night Stroop task with a score ranging from 0 to 12, and age and (c) the statue task with a score ranging from 0 to 30, and age. Each point indicates a score per participant. The shaded area indicates the 95 % confidence intervals of the regression line. A jitter function was applied to make overlapping data points more visible.

~~
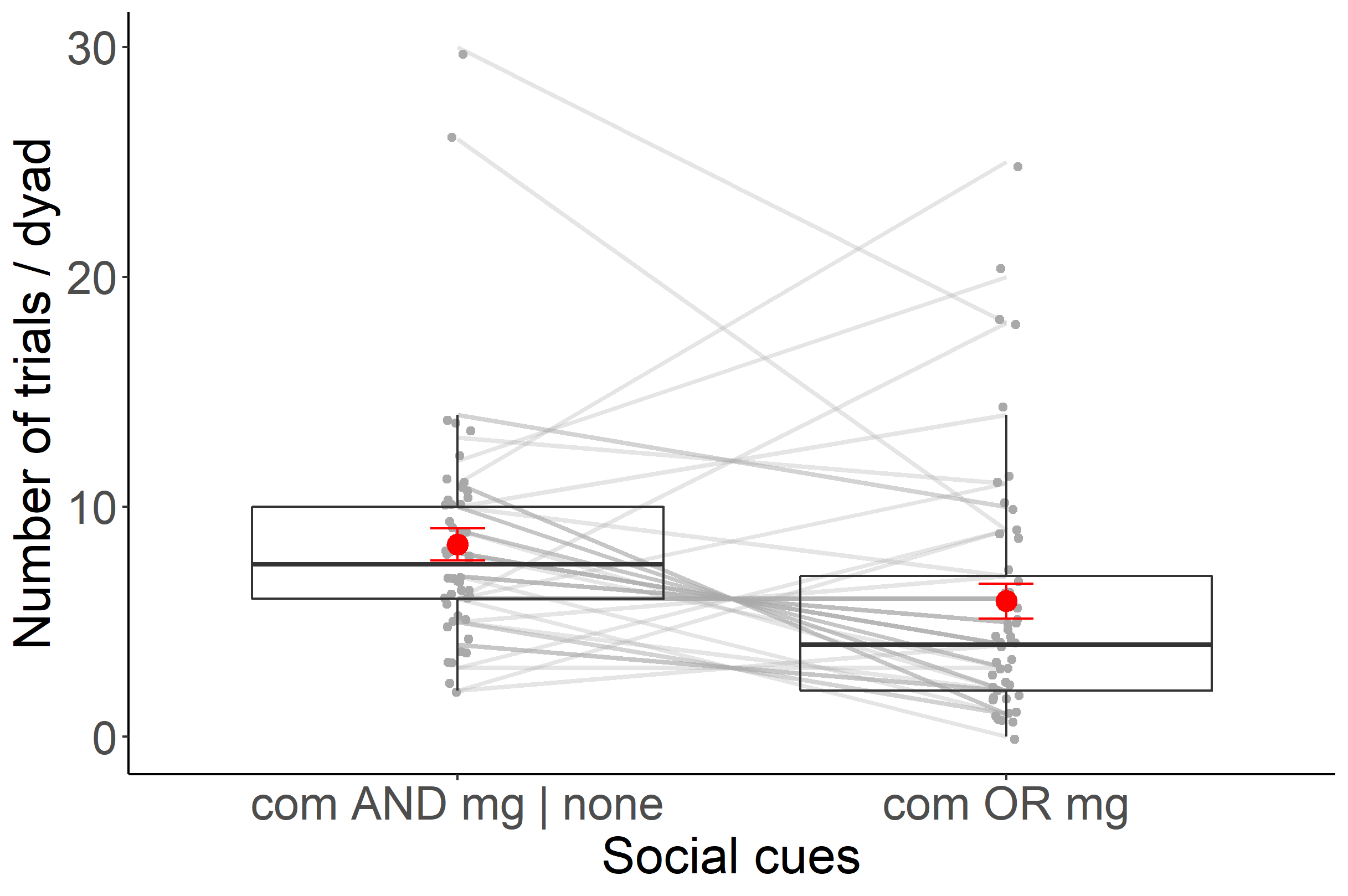
~~

**Figure S8** **Distribution of social cues in the joint task.** Number of trials per dyad containing a communicative cue and a mutual gaze (or none) or one of the social cues but not the other. A jitter function was applied to make overlapping data points more visible. The boxes and whiskers represent medians and lower and upper quartile scores. Error bars (in red) represent standard errors of the mean.





**Figure S9** **Cooperation success in the joint task in the dyads of the four tested species.** Cooperation success was calculated as observed correct response choices resulting in a retrievable reward for both partners per session and species. The boxes and whiskers represent medians and lower and upper quartile scores. Error bars (in red) represent standard errors of the mean.

**Table S1. Simon effect in first heading directions.** Overview of glmm with the participants’ first heading directions (correct or incorrect first movement toward a response side per trial) as the binary dependent variable. Italics: p < 0.05.

| **Fixed factor** | **β** | **SE** | **95% CI** | **z** |  | **p** |
| --- | --- | --- | --- | --- | --- | --- |
| Intercept | 0.90 | 0.50 |  |  |  |  |
| Compatibility | 0.67 | 0.08 | [0.52, 0.82] | 8.62 |  | *< 2 x 10^-16^ **** |
| Task |  |  |  |  |  |  |
| Experimental tasks AC vs Control tasks BD | 0.03 | 0.06 | [-0.08, 0.15] | 0.55 |  | 0.59 |
| Task A vs C | 0.05 | 0.12 | [-0.18, 0.27] | 0.42 |  | 0.68 |
| Task B vs D | 0.07 | 0.10 | [-0.13, 0.27] | 0.67 |  | 0.51 |
| Sex | 0.34 | 0.16 | [0.02, 0.65] | 2.09 |  | *0.04 ** |
| Age | -0.08 | 0.01 | [-0.10, -0.05] | -6.63 |  | *3.39 x 10^-11^**** |
| Session |  |  |  |  |  |  |
| Linear trend | -0.22 | 0.08 | [-0.37, -0.07] | -2.90 |  | *3.68 x 10^-3^ *** |
| Quadratic trend | 0.01 | 0.08 | [-0.14, 0.16] | 0.13 |  | 0.90 |
| Cubic trend | 0.01 | 0.08 | [-0.14, 0.17] | 0.18 |  | 0.86 |
| Sound stimulus | 0.02 | 0.07 | [-0.12, 0.17] | 0.30 |  | 0.76 |
| Side | 0.01 | 0.07 | [-0.14, 0.16] | 0.14 |  | 0.89 |
| Compatibility * Task interactions |  |  |  |  |  |  |
| Compatibility * Task AC vs BD | 0.29 | 0.08 | [0.13, 0.44] | 3.70 |  | *2.15 x 10^-4^ **** |
| Compatibility * Task A vs C | 0.17 | 0.12 | [-0.06, 0.40] | 1.42 |  | 0.15 |
| Compatibility * Task B vs D | -0.02 | 0.10 | [-0.21, 0.18] | -0.15 |  | 0.88 |
| χ^2^_14_ = 196.48, p < 0.001, pseudo-R^2^_c_ = 0.23, N = 4979 of 30 individuals | | | | | | |

**Notes.** A = full task; B = half task; C = joint task; D = joint-control task

**Table S2. Species comparisons of the (joint) Simon effect.** Overview of glmm with the individuals’ first heading directions (correct or incorrect first movement toward a response side per trial) as the binary dependent variable. Italics: p < 0.05.

| **Fixed factor** | **β** | **SE** | **95% CI** | **z** |  | **p** |
| --- | --- | --- | --- | --- | --- | --- |
| Intercept | -0.92 | 0.07 |  |  |  |  |
| Compatibility | 0.86 | 0.05 | [0.76, 0.97] | 16.38 |  | *< 2 x 10^-16^ **** |
| Task |  |  |  |  |  |  |
| Experimental tasks AC vs Control tasks BD | -0.34 | 0.04 | [-0.41, -0.26] | -8.63 |  | *< 2 x 10^-16^ **** |
| Task A vs C | 0.00 | 0.09 | [-0.16, 0.17] | 0.05 |  | 0.96 |
| Task B vs D | 0.00 | 0.08 | [-0.15, 0.16] | 0.04 |  | 0.96 |
| Species |  |  |  |  |  |  |
| Mac & Cap vs Kids & Mar | 0.23 | 0.07 | [0.09, 0.37] | 3.17 |  | *1.53 x 10^-3^ *** |
| Mac vs Cap | 0.02 | 0.12 | [-0.21, 0.24] | 0.16 |  | 0.88 |
| Kids vs Mar | 0.62 | 0.09 | [0.45, 0.79] | 7.24 |  | *4.55 x 10^-13^ **** |
| Compatibility * Task interactions |  |  |  |  |  |  |
| Compatibility * Task AC vs BD | 0.71 | 0.05 | [0.61, 0.81] | 13.49 |  | *< 2 x 10^-16^ **** |
| Compatibility * Task A vs C | 0.01 | 0.08 | [-0.15, 0.17] | 0.14 |  | 0.89 |
| Compatibility * Task B vs D | 0.01 | 0.06 | [-0.11, 0.14] | 0.22 |  | 0.82 |
| Compatibility * Species interactions |  |  |  |  |  |  |
| Compatibility * Mac & Cap vs Kids & Mar | 0.32 | 0.05 | [0.22, 0.42] | 6.07 |  | *1.26 x 10^-9^ **** |
| Compatibility * Mac vs Cap | -0.07 | 0.09 | [-0.25, 0.10] | -0.82 |  | 0.41 |
| Compatibility * Kids vs Mar | -0.10 | 0.06 | [-0.22, 0.01] | -1.86 |  | 0.06 |
| Task * Species interactions |  |  |  |  |  |  |
| Task AC vs BD * Mac & Cap vs Kids & Mar | -0.20 | 0.04 | [-0.28, -0.13] | -5.16 |  | *2.48 x 10^-7^ **** |
| Task A vs C * Mac & Cap vs Kids & Mar | -0.07 | 0.09 | [-0.24, 0.10] | -0.80 |  | 0.42 |
| Task B vs D * Mac & Cap vs Kids & Mar | -0.07 | 0.08 | [-0.22, 0.08] | -0.90 |  | 0.37 |
| Task AC vs BD * Mac vs Cap | 0.02 | 0.07 | [-0.11, 0.15] | 0.35 |  | 0.72 |
| Task A vs C * Mac vs Cap | 0.26 | 0.14 | [-0.02, 0.53] | 1.80 |  | 0.07 |
| Task B vs D * Mac vs Cap | -0.01 | 0.12 | [-0.25, 0.23] | -0.07 |  | 0.95 |
| Task AC vs BD * Kids vs Mar | -0.17 | 0.04 | [-0.25, -0.08] | -3.97 |  | *7.17 x 10^-5^ **** |
| Task A vs C * Kids vs Mar | 0.05 | 0.10 | [-0.14, 0.24] | 0.55 |  | 0.58 |
| Task B vs D * Kids vs Mar | -0.02 | 0.09 | [-0.20, 0.17] | -0.21 |  | 0.83 |
| Compatibility * Task * Species interactions |  |  |  |  |  |  |
| Compatibility * Task AC vs BD * Mac & Cap vs Kids & Mar | 0.34 | 0.05 | [0.24, 0.45] | 6.54 |  | *6.03 x 10^-11^ **** |
| Compatibility * Task A vs C * Mac & Cap vs Kids & Mar | 0.02 | 0.08 | [-0.14, 0.18] | 0.24 |  | 0.81 |
| Compatibility * Task B vs D * Mac & Cap vs Kids & Mar | 0.03 | 0.06 | [-0.10, 0.16] | 0.46 |  | 0.65 |
| Compatibility * Task AC vs BD * Mac vs Cap | 0.00 | 0.09 | [-0.17, 0.17] | -0.01 |  | 1.00 |
| Compatibility * Task A vs C * Mac vs Cap | -0.53 | 0.14 | [-0.82, -0.25] | -3.70 |  | *2.18 x 10^-4^ **** |
| Compatibility * Task B vs D * Mac vs Cap | -0.04 | 0.10 | [-0.24, 0.16] | -0.37 |  | 0.71 |
| Compatibility * Task AC vs BD * Kids vs Mar | 0.09 | 0.06 | [-0.02, 0.20] | 1.58 |  | 0.11 |
| Compatibility * Task A vs C * Kids vs Mar | -0.18 | 0.08 | [-0.34, -0.02] | -2.20 |  | *0.03 ** |
| Compatibility * Task B vs D * Kids vs Mar | 0.00 | 0.08 | [-0.15, 0.16] | 0.04 |  | 0.97 |
| χ^2^_31_ = 565.52, p < 0.001, pseudo-R^2^_c_ = 0.24, N = 10235 of 54 subjects | | | | | | |

**Notes.** A = full task; B = half task; C = joint task; D = joint-control task; Mac = Tonkean macaques; Cap = brown capuchins; Mar = common marmosets

**Table S3. Species comparisons of visual monitoring in the social test conditions.** Overview of glmm with the individuals’ mutual gaze behavior in the joint and the joint-control task as binary dependent variables. Italics: p < 0.05.

| **Fixed factor** | **β** | **SE** | **95% CI** | **z** |  | **p** |
| --- | --- | --- | --- | --- | --- | --- |
| Intercept | -3.41 | 0.32 |  |  |  |  |
| Species |  |  |  |  |  |  |
| Mac & Cap vs Kids & Mar | -1.78 | 0.31 | [-2.39, -1.16] | -5.64 |  | *1.66 x 10^-8^ **** |
| Mac vs Cap | -0.76 | 0.60 | [-1.94, 0.42] | -1.27 |  | 0.21 |
| Kids vs Mar | -0.02 | 0.18 | [-0.38, 0.34] | -0.13 |  | 0.90 |
| Task C vs D | -1.76 | 0.15 | [-2.06, -1.47] | -11.90 |  | *< 2x 10^-16^ **** |
| χ^2^_4_ = 178.41, p < 0.001, pseudo-R^2^_c_ = 0.55, N = 4291 of 53 subjects | | | | | | |

**Notes**. C = joint task; D = joint-control task; Mac = Tonkean macaques; Cap = brown capuchins; Mar = common marmosets
